# Comparison of Sonidegib and Vismodegib Efficiency in Drug-Sensitive and Resistant Ovarian Cancer Cell Lines—2D and 3D Model Study

**DOI:** 10.3390/ijms27219883

**Published:** 2026-11-04

**Authors:** Piotr Stasiak, Justyna Sopel, Artur Płóciennik, Monika Świerczewska, Jan Korbecki, Radosław Januchowski

**Affiliations:** 1 Institute of Biological Sciences, University of Zielona Góra, 65-417 Zielona Góra, Poland; 2 The Doctoral School of Exact and Technical Sciences, University of Zielona Góra, 65-417 Zielona Góra, Poland; 3 Institute of Health Sciences, Collegium Medicum, University of Zielona Góra, 65-417 Zielona Góra, Poland; 4 Department of Histology and Embryology, Poznan University of Medical Sciences, 61-701 Poznań, Poland

**Keywords:** ovarian cancer, multidrug resistance (MDR), sonic hedgehog (Shh) signaling pathway inhibition, Vismodegib, Sonidegib (LDE225), 3D cell culture model

## Abstract

Ovarian cancer is a leading cause of mortality among gynecological malignancies. Despite the initial effectiveness of chemotherapy, multidrug resistance (MDR), driven by ABC transporters such as P-glycoprotein, limits treatment success by reducing intracellular paclitaxel (PAC) accumulation. Abnormalities in the Cancer Stem Cells signaling pathway Sonic Hedgehog (Shh) play a role in drug resistance development and ovarian cancer progression. We evaluated two Shh pathway inhibitors, Sonidegib (LDE225) and Vismodegib (VIS), in the W1 drug-sensitive ovarian cancer cell line and PAC-resistant sublines, using the 2D monolayer model, as well as the 3D spheroid model, to better simulate conditions of the tumor microenvironment. qPCR analysis showed similar levels of Shh pathway gene expression in 2D and 3D cell culture conditions. PAC-resistant cell lines were 3- to 6-fold more sensitive to both LDE225 and VIS than the PAC-sensitive cell line. LDE225 showed a 6- to 10-fold lower IC_50_ than Vismodegib, indicating higher potency. Both agents inhibited proliferation and colony formation in a concentration-dependent manner. Differences in IC_50_ between 2D and 3D dense spheroids formed by the W1 cell line were small, suggesting that high spheroid density does not reduce the activity of LDE225 and VIS against cancer cells. In conclusion, both inhibitors present pleotropic effects against ovarian cancer cells, especially drug-resistant ones, and can be considered as a new treatment opportunity in this cancer. Nevertheless, further investigation of Hedgehog pathway inhibition in more advanced models is required to assess the potential of clinical application for these compounds.

## 1. Introduction

Low effectiveness of chemotherapy is a limiting factor in the treatment of cancer patients. Ovarian cancer is one of the cancers that develop drug resistance during treatment [1,2]. The first-line chemotherapy in ovarian cancer is always composed of platinum-based compounds and taxanes [3]. At the beginning, only five percent of ovarian cancer patients are resistant to chemotherapy; others present different levels of response, from not sensitive to therapy, with recurrence within 6 months after finishing treatment (17%), to partially sensitive to chemotherapy, with recurrence in months 7–12 after treatment (23%), to sensitive, with recurrence 12 months or more after finishing chemotherapy (55%). Unfortunately, only 17% of patients can be designated as cured by chemotherapy, and about 80% eventually develop drug resistance [3].

At the cellular level, the most important role in drug resistance is the expression of drug transporters from the ABC family [4]. Among them, the most significant is glycoprotein P, which can remove over 20 cytotoxic drugs from cancer cells, among them paclitaxel (PAC) used in ovarian cancer chemotherapy [4,5]. We and others observed overexpression of P-Glycoprotein (P-gp) in PAC-resistant ovarian cancer cell lines [6,7,8]. Overexpression of P-gp is also related to lower progression-free survival in ovarian cancer patients [9]. Increased resistance to chemotherapy also seems to be related to increased protein phosphorylation and stronger signal transduction [10] and was observed during drug resistance development in cancer and cancer cell lines [11,12].

The next level of resistance is related to the cancer microenvironment [13]. Tumor cells and cancer-associated fibroblasts (CAFs) can produce extracellular matrix (ECM) components that play a passive and active role in drug resistance. Some drugs, like PAC and methotrexate (MTX), can directly bind to ECM molecules, limiting their accessibility to cancer cells [14]. The interaction of ECM with receptors on cancer cells can activate signal transduction pathways leading to increased expression of drug resistance genes and increased resistance to apoptosis in the mechanism designated as Cell Adhesion Mediated Drug Resistance (CAM-DR) [13,15]. Increased expression of many ECM components was observed by us in drug-resistant ovarian cancer cell lines [16,17,18] and played a role in CAM-DR [19,20]. As a tumor is not a single cell but a complex tissue-like structure composed of tumor cells and CAFs, a dense cellular structure and the presence of ECM can be a very effective barrier to the delivery of some cytotoxic drugs like PAC or MTX to tumor cells [14,21,22]. Dense spheroid structure also very effectively limits PAC, vincristine (VIN), and MTX efficiency [23].

According to the CSCs model of drug resistance development, the development of drug resistance is associated with the presence of CSCs in the tumors [24]. A characteristic feature of CSCs is the high expression level of ABC transporters and other protective mechanisms, causing resistance to chemo- and radiotherapy [25,26]. Aldehyde dehydrogenase isoform 1A1—ALDH1A1 is the most common marker of CSCs among solid tumors [24], including ovarian cancer [27,28]. The high number of ALDH1-positive cells negatively correlates with an ovarian cancer patient’s survival [29,30]. The presence of ALDH1A1-positive cells in drug-resistant ovarian cancer cell lines was observed in many studies [29,31,32]. Previously, we also observed ALDH1A1-positive cell populations in PAC and TOP-resistant ovarian cancer cell lines [17,33]. Furthermore, these ALDH1A1+ cells expressed ECM molecules, especially collagens, suggesting that CSCs are responsible for ECM production [17,34].

The biology of cancer stem cells is regulated by different signaling pathways, including Wnt/β-catenin, Notch, and Sonic Hedgehog (Shh) [35]. Although these pathways are called “CSCs signaling pathways”, their activity in cancer cells is not strictly limited to CSCs, but also occurs in other cell populations, particularly in drug-resistant cells. Among CSCs’ signaling pathways, the Shh pathway seems to be the most important. It has been reported that the Shh signaling pathway was active in both ALDH1A1+ (CSCs) and ALDH1A1− cancer cell populations, and targeting this pathway has increased sensitivity to chemotherapy in both [36].

Secretion of the Shh ligand results in its binding to the PTCH1 protein present in the plasma membrane, causing the relocation of the SMO protein, otherwise inhibited by PTCH1. Next, SMO activates downstream signal transduction, including the dissociation of GLI (glioma-associated oncogene) proteins (the transcriptional effectors of the Shh pathway) from the protein complex containing GSK3β (glycogen synthase kinase-3) and, among others, the key intracellular Shh pathway regulator SUFU. This releases the GLI family transcription factors, which then translocate to the nucleus and regulate gene transcription, including, among others, *MDR1* and *BCRP*, the genes involved in drug resistance [37,38].

Abnormalities in Shh pathway activity play a significant role in the development of many cancers [39,40], causing metastasis and drug resistance [41], and have been described in cancers such as colon, bladder, lung, prostate, and ovarian cancer [41]. For these reasons, this pathway seems to be an attractive target in cancer therapy.

Among many inhibitors of the Shh pathway, the SMO protein inhibitors Sonidegib and Vismodegib seem to be the most significant.

Sonidegib, also known as Erismodegib or LDE225, was approved by the FDA in 2015 for treatment of basal cell carcinoma (BBC) and is currently used in clinical trials to treat solid tumors and hematological malignancies [38]. In cell line studies, it has been observed that LDE225 induces cell cycle arrest and apoptosis [42]. LDE225 also effectively inhibited EMT and invasive potential of cancer cells in glioblastoma, as well as prostate and renal cell carcinomas, indicating that it can target both CSCs and non-CSCs populations [43,44,45]. LDE225 was also tested in a Phase I clinical trial in combination with paclitaxel in various solid tumors, including 9 ovarian cancer patients, 5 of whom were considered resistant to taxane chemotherapy; 2 of the latter group achieved stable disease following SMO treatment [46]. However, this clinical trial was designed to assess safe and recommended doses of the drug combination and not to verify whether LDE225 is capable of reversing drug resistance [46].

Vismodegib (VIS) was approved by the FDA in 2012 as the first Shh pathway drug for cancer treatment. Currently, it is used for the treatment of metastatic BCC patients, BCC patients with recurrence, or patients not classified for surgery [47]. VIS is also currently investigated in monotherapy or in combination in many clinical trials, including acute myeloid leukemia, metastatic pancreatic cancer, metastatic prostate cancer, recurrent glioblastoma, and small cell lung cancer [38]. In a mouse xenograft study, VIS treatment caused apoptosis and reduced the tumor volume of human prostate cancer [48]. In breast cancer cells, VIS treatment resulted in cell apoptosis, reduced proliferation, invasion, and mammosphere formation [49]. Moreover, VIS was used in Phase II clinical trials as a possible maintenance therapy in ovarian cancer patients in second or third remission; however, a clinically meaningful improvement was not noted [50].

As far as we know, the Shh pathway inhibitors have not been used in combination with chemotherapeutic drugs to reverse ovarian cancer chemoresistance. Thus, in our study, we wanted to investigate and compare the effects of LDE225 and VIS on the drug-sensitive and resistant ovarian cancer cell lines. Here, we used drug-sensitive W1 cell lines and their derivatives resistant to PAC: W1PR1 and W1PR2 cell lines [33,34]. Additionally, we used the W1PR1-C7 subline with knockdown of ALDH1A1 gene expression [51]. Drug-resistant cell lines are characterized by overexpression of P-gp [33,34], increased protein phosphorylation [12,34], presence of ALDH1A1^+^ cells [17,33,34], and production of ECM components [16,17,18]. Additionally, these cell lines form different kinds of spheroids under non-adhesive conditions [23].

Our cell lines allow us to simulate the chemotherapy resistance mechanisms at a single-cell level in a 2D model, as well as at the level of 3D spheroids that much better reflect real tumor conditions [52]. As both sensitive and resistant cell lines grow in both cell culture conditions, our model seems to be very attractive for investigating the efficiency of SMO inhibitors in ovarian cancer.

## 2. Results

### 2.1. Drug-Resistant Genes and Protein Expression in the Investigated Cell Lines

At the beginning of our study, we performed experiments to compare the expression of the investigated genes and proteins in the cell lines used in our study.

#### 2.1.1. Analysis of ALDH1A1 Expression

**Figure 1:**
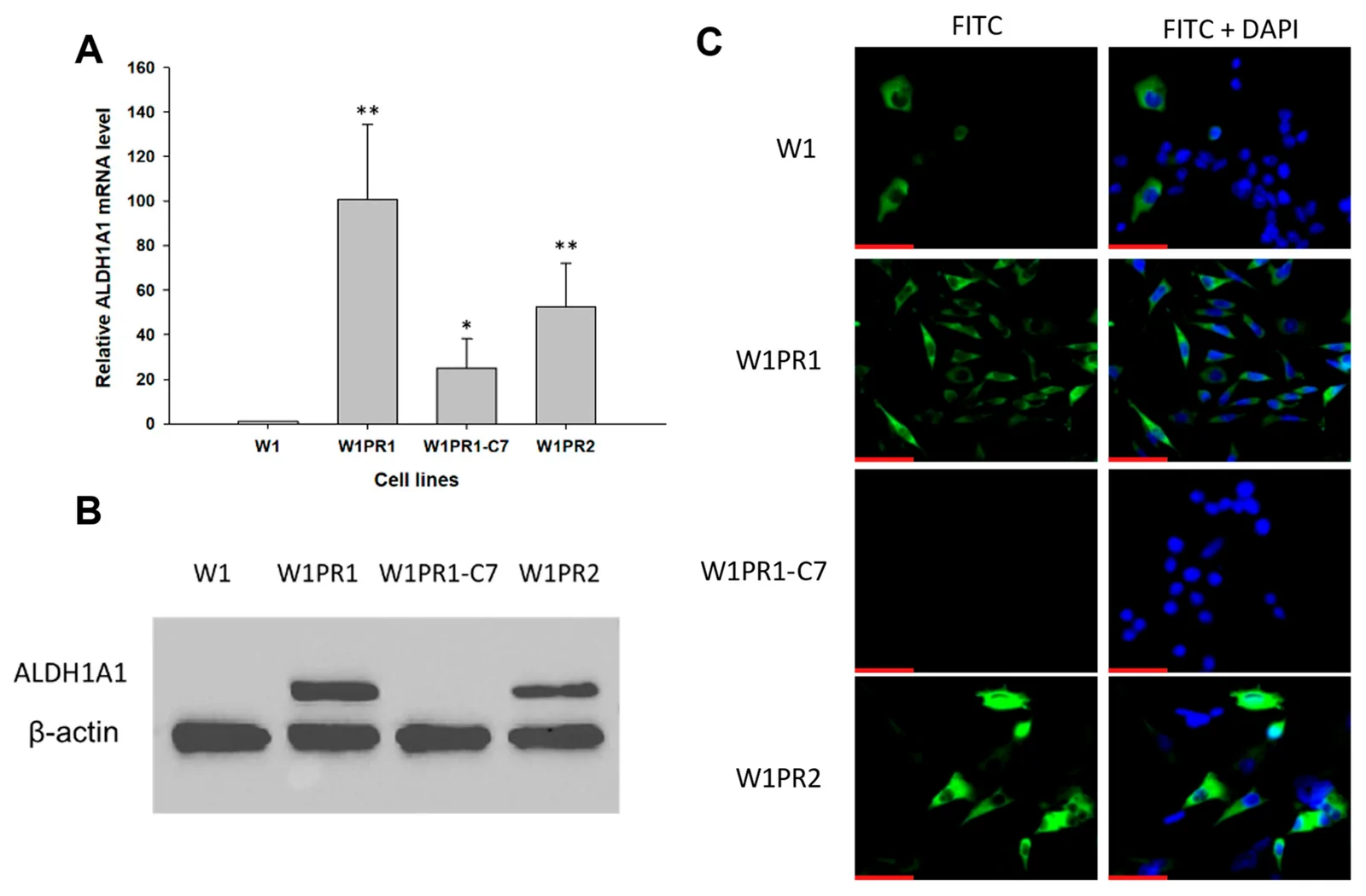
ALDH1A1 expression in W1 cells and paclitaxel-resistant sublines. (**A**) Relative ALDH1A1 transcript levels measured by qPCR (mean from at least 3 independent experiments). Expression in resistant cells was normalized to the parental W1 cell line (value = 1). Statistical significance was assessed using Student’s *t*-test, with * *p* < 0.05 and ** *p* < 0.01 considered statistically significant. (**B**) Western blot analysis of ALDH1A1 protein expression. β-actin served as a loading control. (**C**) Representative immunofluorescence images showing ALDH1A1 expression (green) and nuclear staining with DAPI (blue). Scale bar = 50 μm.

Expression of ALDH1A1, a marker of CSCs, was investigated. Analysis of quantitative polymerase chain reaction (qPCR) results showed about a 100-fold increase in ALDH1A1 transcript levels in the W1PR1 cell line and about a 50-fold increase in the W1PR2 cell line (*p* < 0.01). We also observed a 20-fold increase in ALDH1A1 transcript levels in the W1PR1-C7 cell line with ALDH1A1 gene knockdown (Figure 1A). Western blot analysis confirmed the presence of ALDH1A1 protein in W1PR1 and W1PR2 cell lines and its absence in W1 and W1PR1-C7 cell lines (Figure 1B). Immunofluorescence analysis showed singular cells with ALDH1A1 expression in W1 cell lines and confirmed expression in all cells within the W1PR1 cell line. In contrast, in W1PR2 cell lines, two cell populations could be distinguished. ALDH1A1+ cells with very strong ALDH1A1 expression and ALDH1A1− cells with no ALDH1A1 expression. Knockdown of the ALDH1A1 gene in the W1PR1-C7 cell line caused a lack of ALDH1A1-positive cells in the W1PR1-C7 cell line (Figure 1C).

#### 2.1.2. Analysis of MDR1/P-gp Expression

**Figure 2:**
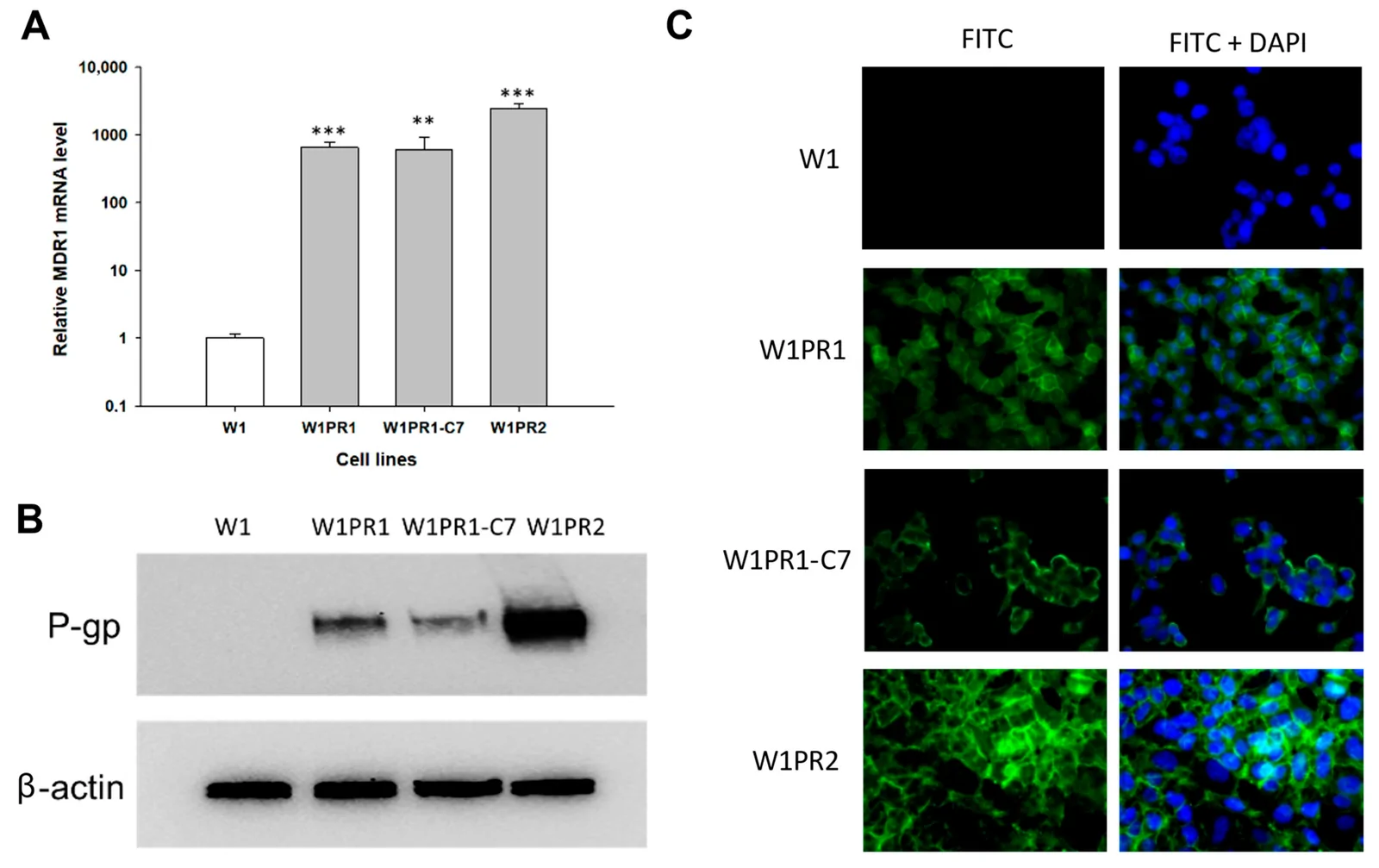
MDR1/P-gp expression in W1 cells and paclitaxel-resistant sublines. (**A**) Relative MDR1 transcript levels measured by qPCR (mean from at least 3 independent experiments). Expression in resistant cells was normalized to the parental W1 cell line (value = 1). Statistical significance was assessed using Student’s *t*-test, with ** *p* < 0.01 and *** *p* < 0.001 considered statistically significant. (**B**) Western blot analysis of P-gp protein expression. β-actin served as a loading control. (**C**) Representative immunofluorescence images showing P-gp expression (green) and nuclear staining with DAPI (blue). Scale bar = 50 μm.

P-gp is the most important drug transporter responsible for PAC resistance [4,5]. Thus, we analyzed the expression of the *MDR1* gene and P-gp protein in cell lines used in this study. The qPCR results showed a tremendous increase in *MDR1* transcript level in all PAC-resistant cell lines. Specifically, we observed about a 1000-fold increase in *MDR1* transcript level in W1PR1 (*p* < 0.001) and W1PR1-C7 cell lines (*p* < 0.01) and over a 2000-fold increase in the W1PR2 cell line (*p* < 0.001) (Figure 2A). In Western blot analysis, we did not observe P-gp expression in the W1 drug-sensitive cell line; however, the bands corresponding to P-gp protein were observed in all PAC-resistant cell lines, with expression levels matching transcript levels (Figure 2B). Immunofluorescence analysis confirmed P-gp expression in the cell membrane of PAC-resistant cell lines (Figure 2C).

#### 2.1.3. Analysis of COL3A1 Expression

**Figure 3:**
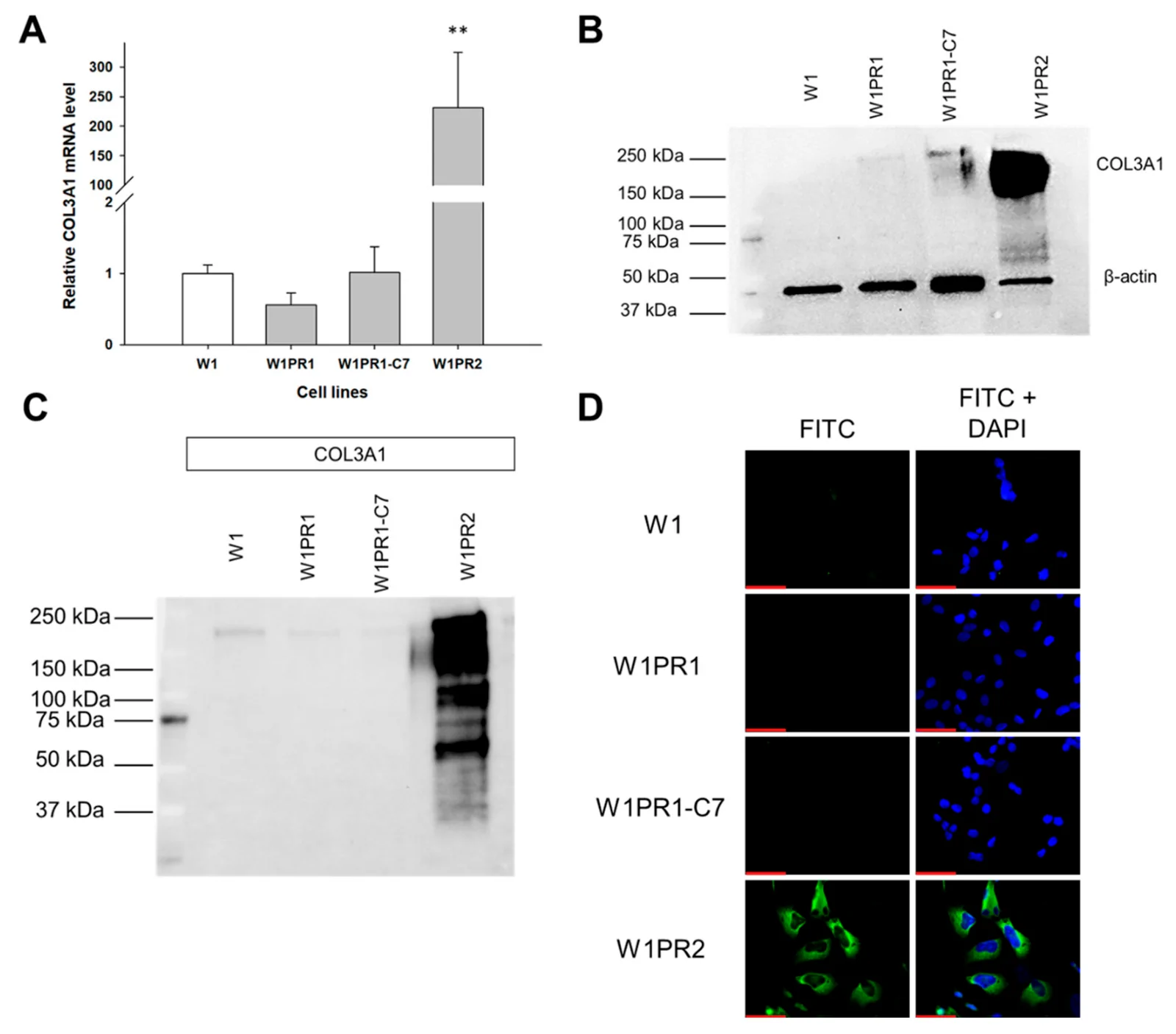
COL3A1 expression in W1 cells and paclitaxel-resistant sublines. (**A**) Relative COL3A1 transcript levels measured by qPCR (mean from at least 3 independent experiments). Expression in resistant cells (grey bars) was normalized to the parental W1 cell line (value = 1; white bar). Statistical significance was assessed using Student’s *t*-test, with ** *p* < 0.01 considered statistically significant. (**B**) Western blot analysis of intracellular COL3A1 protein expression. β-actin served as a loading control. (**C**) Western blot analysis of extracellular COL3A1 protein expression. (**D**) Representative immunofluorescence images showing COL3A1 expression (green) and nuclear staining with DAPI (blue). Scale bar = 50 μm.

Expression of COL3A1, a protein related to CAM-DR, was also investigated. qPCR analysis showed similar levels of COL3A1 transcript in W1, W1PR1, and W1PR1-C7 cell lines. In contrast, in the W1PR2 cell line, we observed about a 200-fold increase in COL3A1 transcript level (*p* < 0.01) (Figure 3A). Western blot analysis showed an intense COL3A1 band corresponding to about 200 kDa only in lysates from W1PR2 cell lines (Figure 3B). Analysis of COL3A1 protein in cell culture medium confirmed the presence of COL3A1 protein only in cell culture medium derived from the W1PR2 cell line, with bands from 60 to about 250 kDa (Figure 3C). Also, the immunofluorescence analysis showed the presence of COL3A1 protein only in the W1PR2 cell line (Figure 3D).

#### 2.1.4. Expression Analysis of pTYR

**Figure 4:**
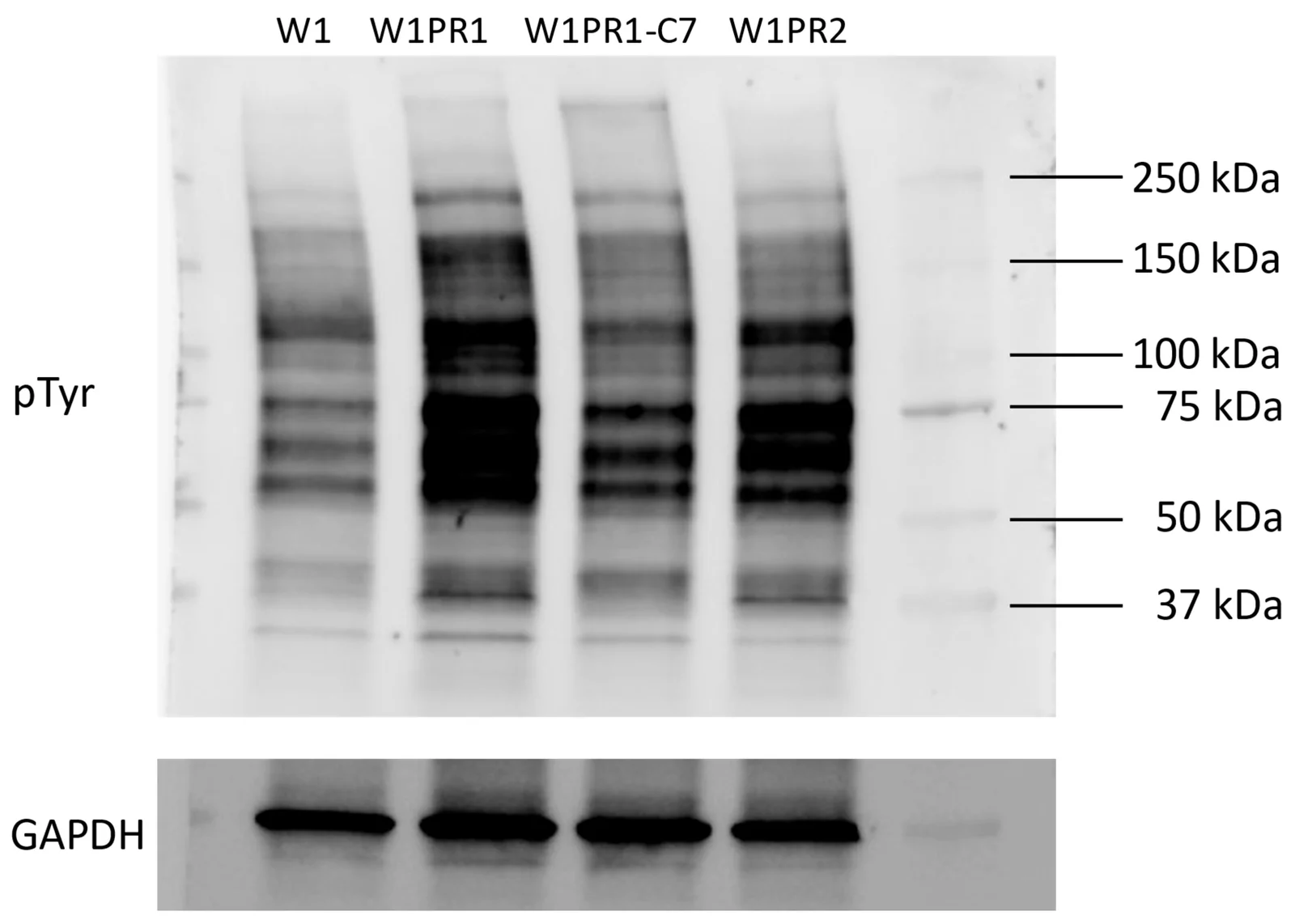
Total pTYR protein expression in W1 and paclitaxel-resistant sublines. Protein levels were assessed via Western blot analysis in cells cultured as a monolayer. GAPDH served as the loading control.

As drug resistance is often related to increased signal transduction, we also compared the levels of total tyrosine phosphorylation (pTyr) in the investigated cell lines. Western blot analysis showed a strong increase in total pTyr levels in PAC-resistant cell lines, especially in W1PR1 and W1PR2 cell lines (Figure 4).

#### 2.1.5. Analysis of the Expression of PCTCH1, SHH, and SMO Genes

**Figure 5:**
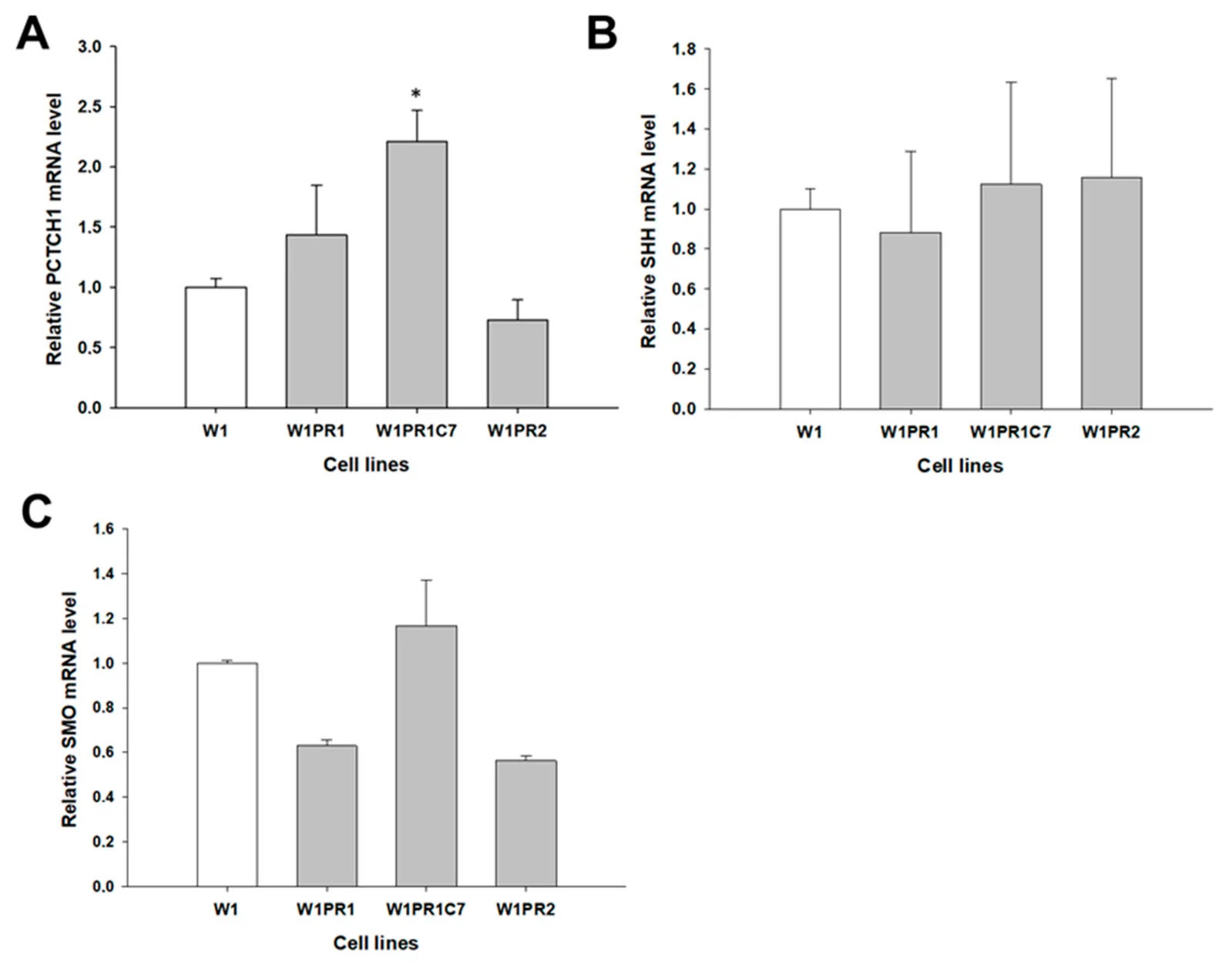
qPCR analysis of PCTCH1 (**A**), SHH (**B**), and SMO (**C**) gene expression in W1 and paclitaxel-resistant sublines (mean from at least 3 independent experiments). Relative expression levels in resistant cells (gray bars) were calculated in comparison with the parental W1 cell line (set as 1; white bar). Statistical significance was assessed using Student’s *t*-test, with * *p* < 0.05 considered statistically significant.

Next, we checked the expression of *PCTCH1*, *SHH*, and *SMO* genes involved in the SHH signaling pathway. The analysis showed only about a 2-fold increase in *PCTCH1* transcript level in the W1PR1-C7 cell line (*p* < 0.05) (Figure 5A). However, we did not observe statistically significant differences in *SHH* and *SMO* transcript levels among the investigated cell lines (Figure 5B,C).

#### 2.1.6. Analysis of GLI-1 and GLI-2 Expression

**Figure 6:**
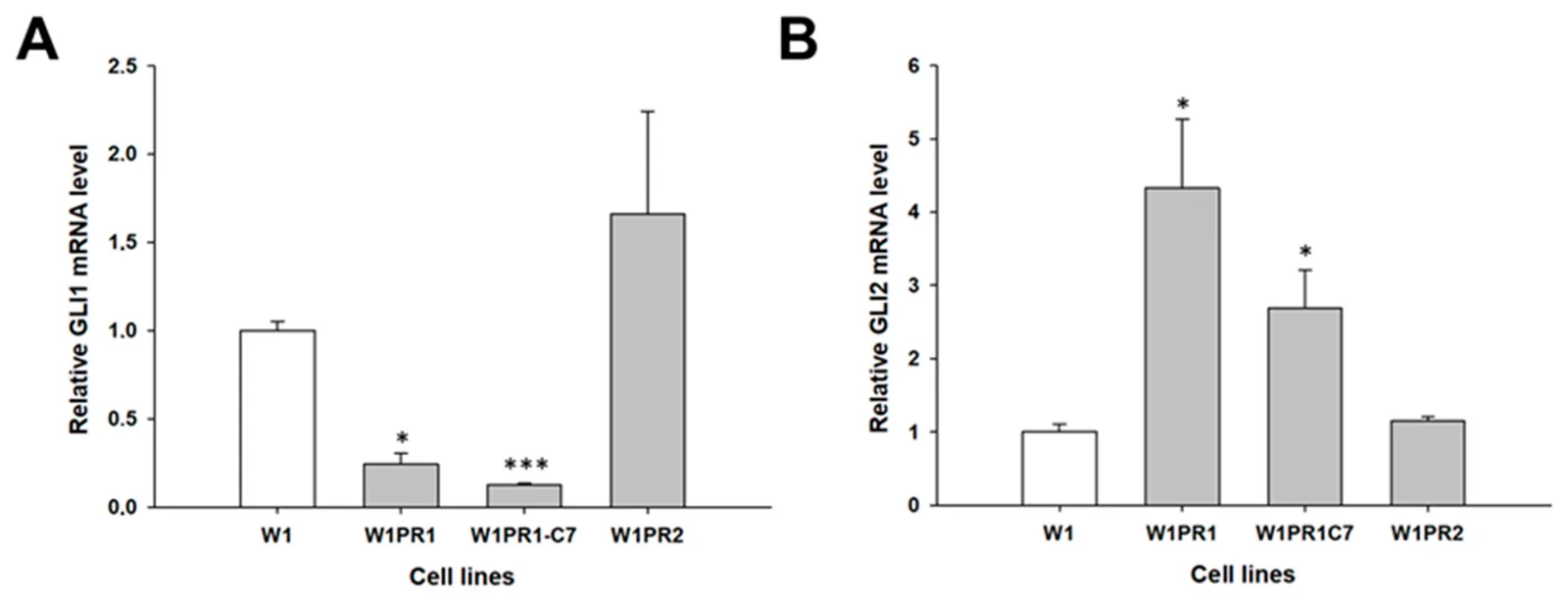
qPCR analysis of GLI1 (**A**) and GLI2 (**B**) gene expression in W1 and paclitaxel-resistant sublines (mean from at least 3 independent experiments). Relative expression levels in resistant cells (grey bars) were calculated in comparison with the parental W1 cell line (set as 1; white bar). Differences were considered statistically significant at * *p* < 0.05, *** *p* < 0.001.

Next, we investigated the expression of GLI transcription factors. Analysis of *GLI-1* transcript showed about a 2-fold decrease in the expression level in the W1PR1 cell line (*p* < 0.05) and about a 3-fold decrease in the W1PR1-C7 cell line (Figure 6A). In contrast, we observed an increase in GLI-2 transcript levels in the W1PR1 cell line (4-fold, *p* < 0.05) and W1PR1-C7 (3-fold, *p* < 0.05) (Figure 6B).

### 2.2. The Response of Drug-Sensitive and Resistant Cell Lines to LDE225 Treatment in the 2D Model

**Figure 7:**
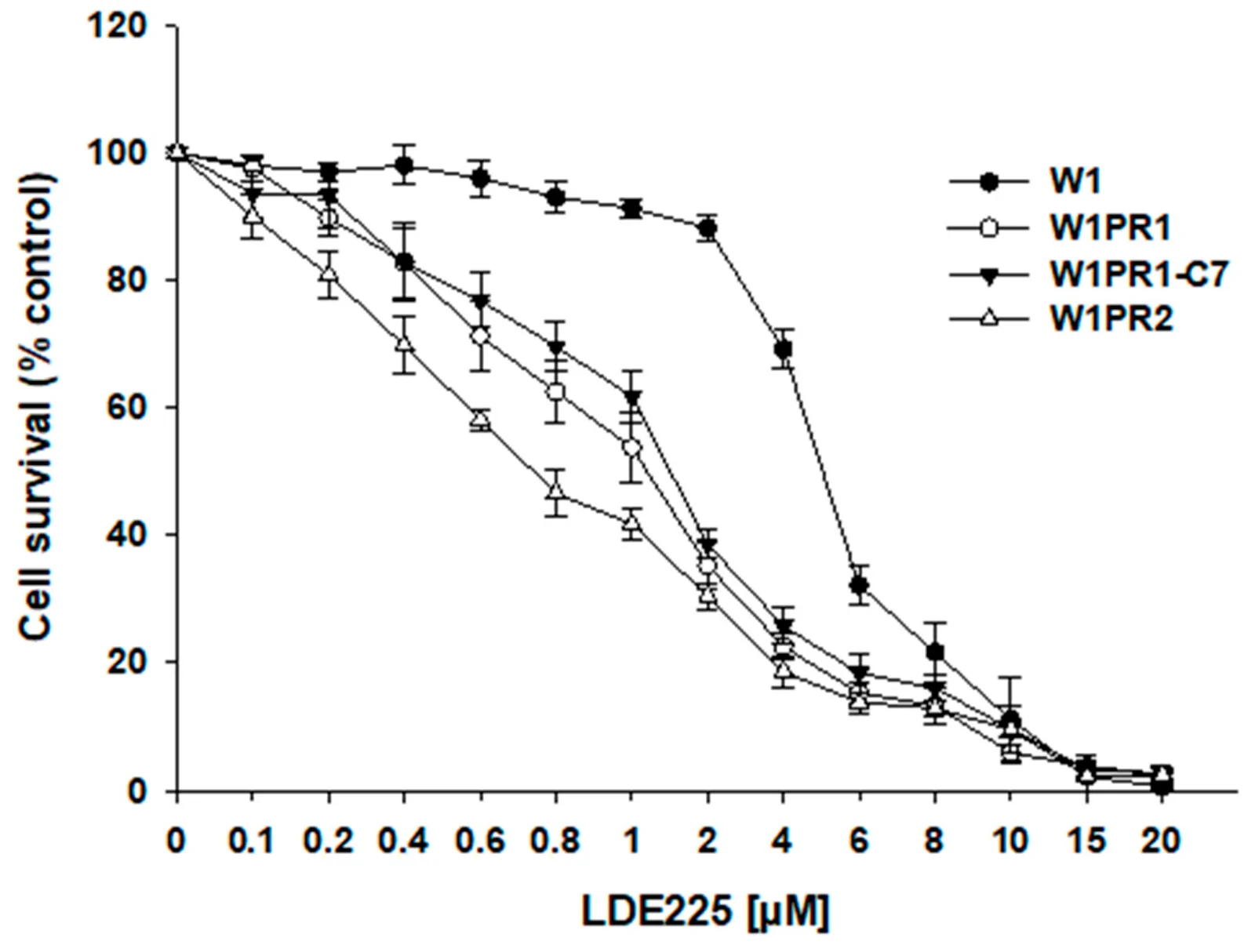
MTT assay of W1 and paclitaxel-resistant sublines, following LDE225 treatment (mean from at least 4 independent experiments). Cell viability was evaluated using the MTT assay after 72 h of exposure to increasing concentrations of LDE225. Results are presented as mean ± SEM and normalized to the untreated control.

The effect of the SHH pathway inhibitor (specifically, an SMO protein inhibitor) Sonidegib—also known as LDE225—on the viability of ovarian cancer cell lines was investigated. We observed very different responses to LDE225 treatment when we compared the drug-sensitive W1 cell line and the PAC-resistant cell lines. The W1 cell line indicated no sensitivity to low concentrations of LDE225 (up to 2 µM). However, a higher increase in LDE225 concentration resulted in a rapid concentration-dependent increase in cell sensitivity. In contrast, the PAC-resistant cell lines were sensitive to LDE225 concentration starting from 0.1 µM with a linear concentration-dependent response curve. A similar level of response was observed in W1PR1 and W1PR1-C7 cell lines. The most sensitive was the W1PR2 cell line (Figure 7). Based on the response curves, the IC_25_ and IC_50_ values of LDE225 were calculated (Table 1). In PAC-resistant cell lines, the IC_25_ values were lower compared to the W1 cell line. Specifically, in the W1PR1 cell line, IC_25_ value was 0.52 µM vs. 3.39 µM in the W1 cell line and it decreased 6.62-fold (*p* < 0.001); in W1PR1-C7, IC_25_ value was 0.66 µM vs. 3.39 µM in the W1, and it decreased 5.13-fold (*p* < 0.001); in W1PR2, the IC_25_ value was 0.31 µM vs. 3.39 µM in the W1 cell lines and it decreased 10.3-fold (*p* < 0.001). Similarly, IC_50_ values were 1.27 µM in the W1PR1 cell line vs. 5.03 µM in the W1 cell line, and it decreased 3.96-fold (*p* < 0.001); 1.45 µM in the W1PR1-C7 cell line vs. 5.03 µM in the W1 cell line and it decreased 3.47-fold (*p* < 0.001); 0.76 µM in the W1PR2 cell line vs. 5.03 µM in the W1 cell line, and it decreased 6.62-fold (*p* < 0.001).

### 2.3. The Response of Drug-Sensitive and Resistant Cell Lines to VIS Treatment in the 2D Model

**Figure 8:**
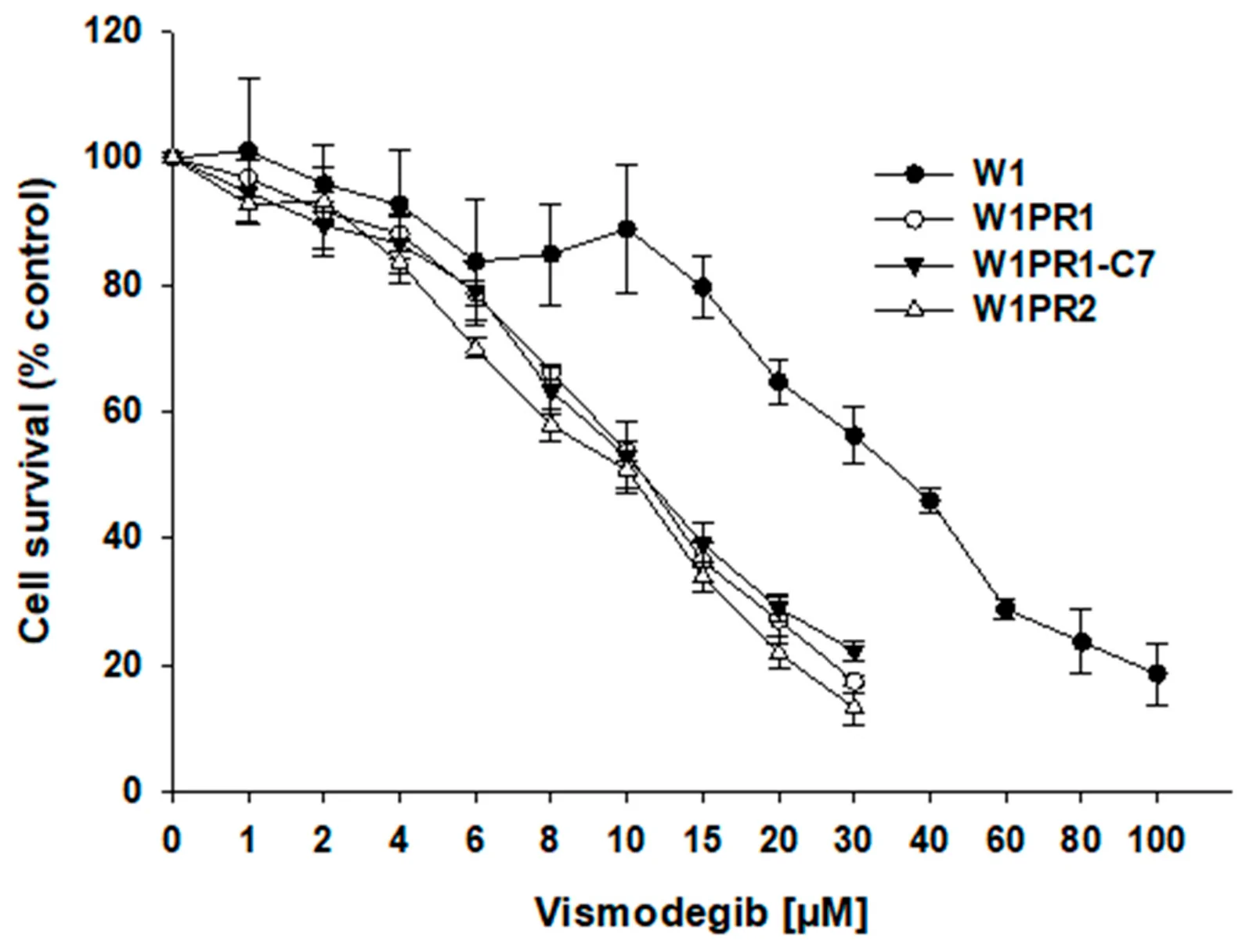
MTT assay of W1 and paclitaxel-resistant sublines, following Vismodegib treatment (mean from at least 4 independent experiments). Cell viability was evaluated using the MTT assay after 72 h of exposure to increasing concentrations of LDE225. Results are presented as mean ± SEM and normalized to the untreated control.

We also investigated the effect of the second inhibitor of the SMO protein—Vismodegib (VIS). In the W1 cell line, we observed a minor effect of VIS at concentrations up to 10 µM. A higher increase in VIS concentration resulted in a concentration-dependent decrease in cell viability. PAC-resistant cell lines showed a concentration-dependent response; however, they were much more sensitive than the W1 cell line (Figure 8). Based on the cellular response, we calculated the IC_25_ and IC_50_ values (Table 2). In PAC-resistant cell lines, the IC_25_ value was lower than in the W1 cell line. Specifically, in the W1PR1 cell line, the IC_25_ value was 6.50 µM vs. 17.33 µM in the W1 cell line, and it decreased 2.67-fold (*p* < 0.01); in the W1PR1-C7 cell line the IC_25_ value was 6.44 µM vs. 17.33 µM in W1 cell line and it decreased 2.69-fold (*p* < 0.01); in the W1PR2 cell line the IC_25_ value was 5.19 µM vs. 17.33 µM in the W1 cell lines and it decreased 3.34-fold (*p* < 0.01). Similar differences were observed in the case of the following IC_50_ values: 11.1 µM in the W1PR1 cell line vs. 33.90 µM in the W1 cell line—it decreased 3.05-fold (*p* < 0.001); 11.1 µM in the W1PR1-C7 cell line vs. 33.90 µM in the W1 cell line—it decreased 3.05-fold (*p* < 0.001); 10.3 µM in the W1PR2 cell line vs. 33.90 µM in the W1—it decreased 3.29-fold (*p* < 0.001).

### 2.4. The Effect of Co-Treatment with SMO Inhibitors and PAC on Cell Viability

As a next step, we investigated whether treatment with both SMO protein inhibitors would increase sensitivity to PAC in the PAC-resistant cell lines. All cell lines were treated with increasing concentrations of PAC with or without LDE225 or VIS at concentrations close to IC_10_, IC_25_, and IC_50_.

**Figure 9:**
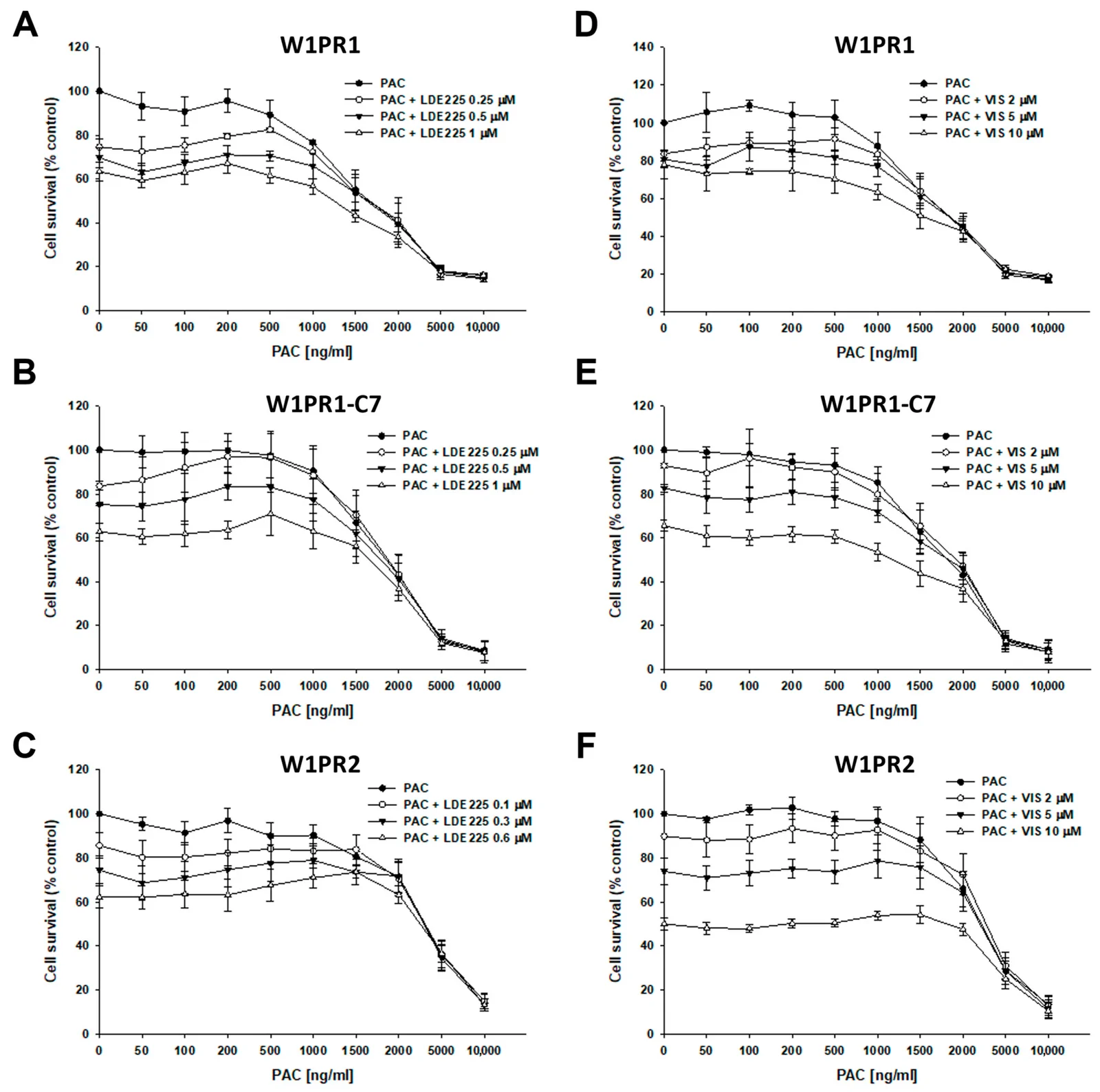
Effect of SMO inhibitors (LDE225, VIS) on PAC-resistant cell lines. Effect of LDE225 on W1PR1 (**A**), W1PR1-C7 (**B**), and W1PR2 (**C**), and effect of VIS on W1PR1 (**D**), W1PR1-C7 (**E**), and W1PR2 (**F**). Cells were treated with paclitaxel (PAC) alone or in combination with LDE225 (0.25, 0.3, 0.5, 0.6, or 1 μM) or Vismodegib (2, 5, or 10 μM) for 72 h. Cell viability was assessed using the MTT assay. Results are presented as mean ± SEM and normalized to the untreated control.

Treatment with LDE225 alone did not increase sensitivity to PAC in any of the cell lines investigated. Decreased viability of the cells grown at lower PAC concentrations was caused only by LDE225. At higher PAC concentrations, cell viability was very similar, regardless of LDE225 presence (Figure 9A–C), resulting in similar IC_50_ values (Table 3).

A comparable response was noted in cells co-treated with VIS and PAC. Vismodegib-treated cells were not more sensitive to PAC. The lower viability of the cells grown in low PAC concentration was caused only by VIS treatment. At higher PAC concentrations, the viability of cells was similar regardless of VIS presence (Figure 9D–F), which was reflected by similar IC_50_ values. Only at the VIS concentration of 10 µM, we noted a decrease in the IC_50_ value for PAC. However, these differences were not statistically significant (Table 4).

### 2.5. The Effect of SMO Inhibitors on the Drug Resistance Mechanisms

We tested whether treatment with SMO inhibitors would change the expression of resistance proteins in the investigated cell lines. Thus, we checked the expression of selected proteins after 72 h of treatment, corresponding to the time of MTT experiments. We investigated the effect of inhibitors at concentrations of IC_25_ and IC_50_.

#### 2.5.1. Effect of SMO Inhibitors on P-gp Protein Expression

**Figure 10:**
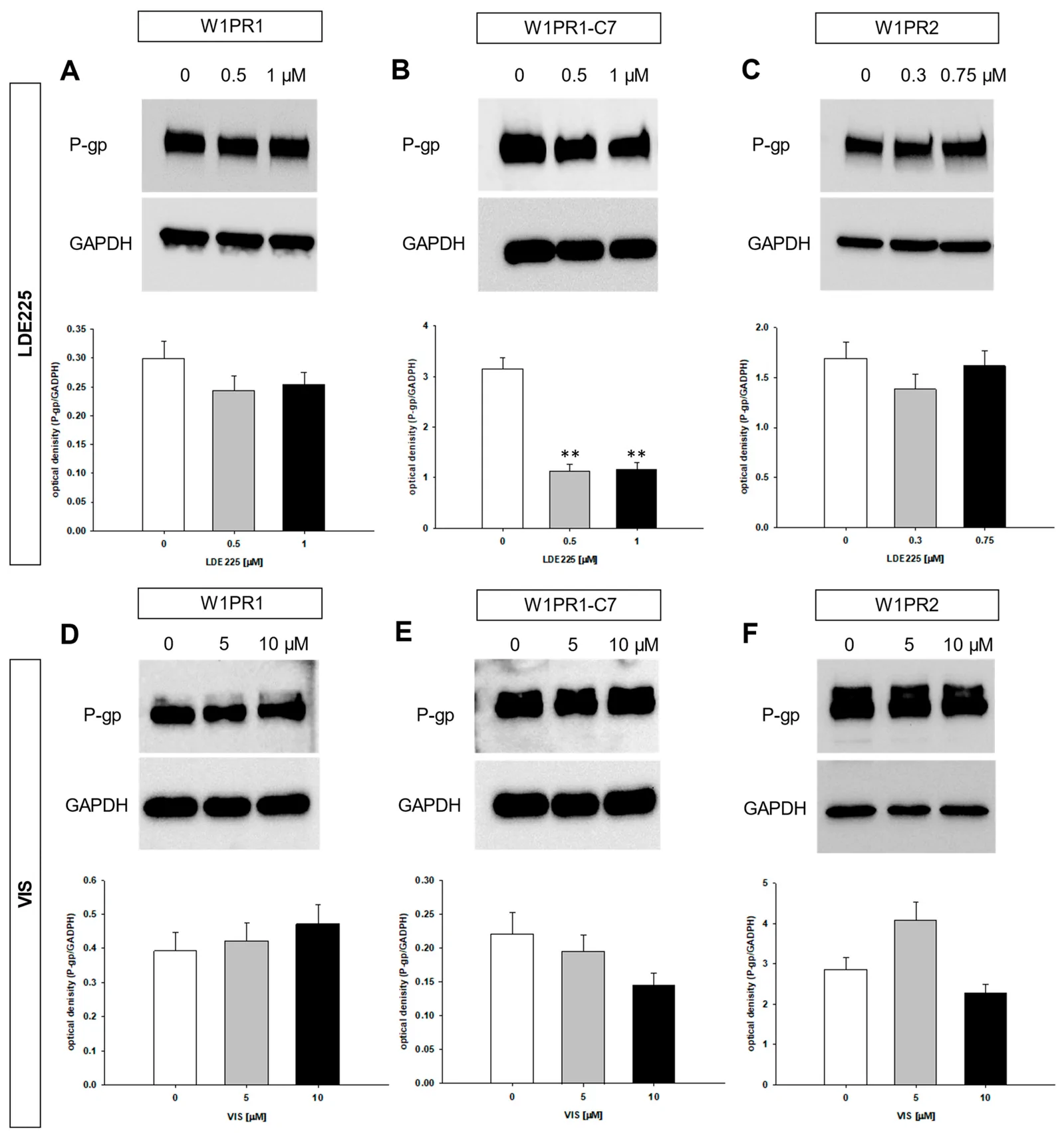
Effect of LDE225 (**A**–**C**) and Vismodegib (**D**–**F**) on P-gp protein expression in paclitaxel-resistant cell lines. P-gp protein expression was analyzed in W1PR1 (**A**,**D**), W1PR1-C7 (**B**,**E**), and W1PR2 (**C**,**F**) cells following treatment with LDE225 or Vismodegib under monolayer culture conditions. Protein levels were assessed via Western blot analysis. GAPDH served as the loading control. Densitometric measurements are presented in charts below the corresponding blots. Statistical significance was assessed using Student’s *t*-test, with ** *p* < 0.01 considered statistically significant.

As P-gp plays the most significant role in paclitaxel resistance, we verified the expression of this protein in W1PR1, W1PR1-C7, and W1PR2 cell lines after LDE225 and VIS treatments. Western blot results are summarized in Figure 10. No effect on P-gp protein expression was observed following either LDE225 treatment in W1PR1 and W1PR2 cell lines (Figure 10A,C), or VIS treatment in all cell lines (Figure 10D–F). Decreased P-gp protein level was observed in W1PR1-C7 cell line after LDE225 treatment *p* < 0.05 (Figure 10B).

#### 2.5.2. Effect of SMO Inhibitors on ALDH1A1 Protein Expression

**Figure 11:**
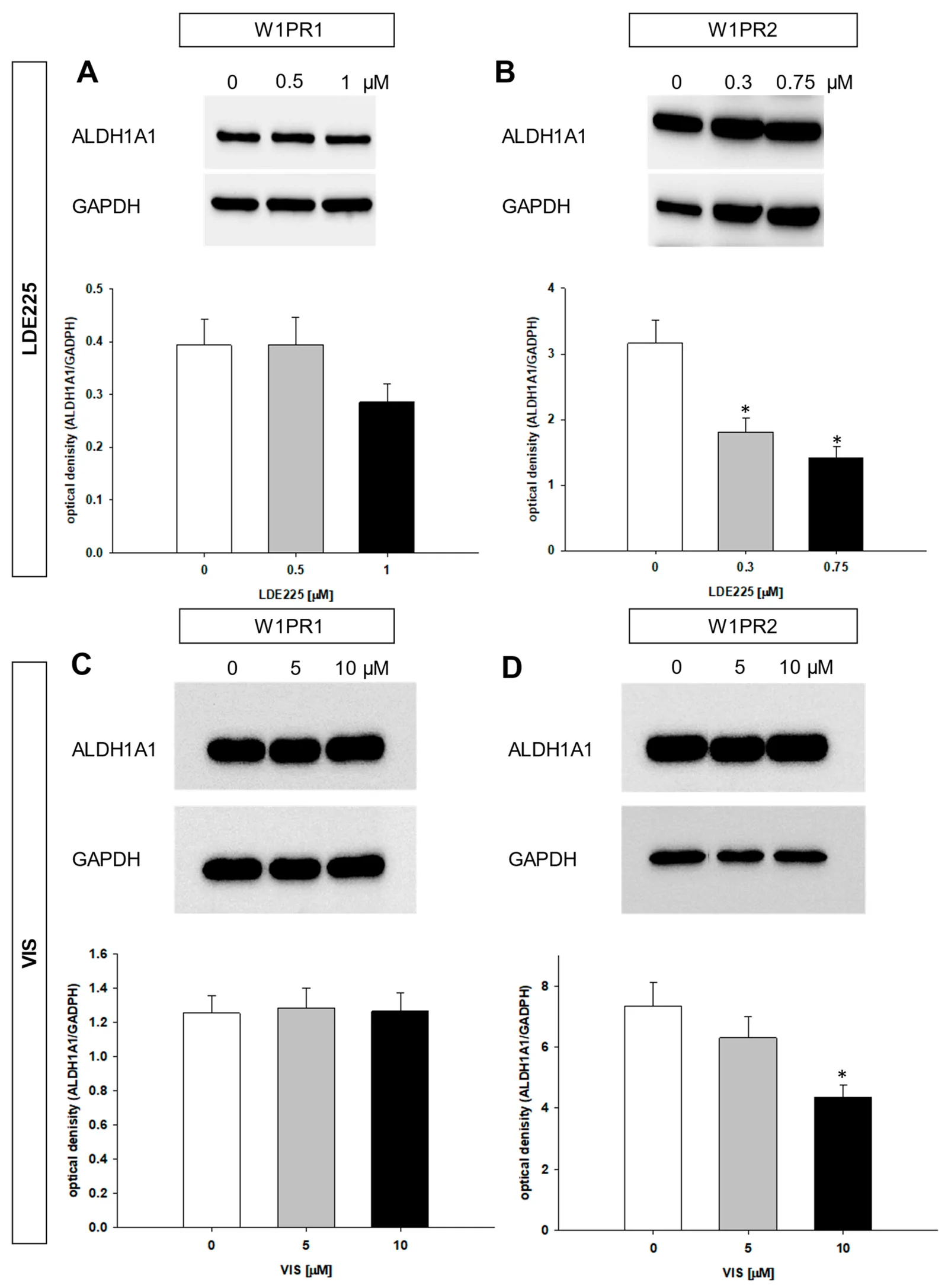
Effect of LDE225 (**A**,**B**) and Vismodegib (**C**,**D**) on ALDH1A1 protein expression in paclitaxel-resistant cell lines. ALDH1A1 protein expression was analyzed in W1PR1 (**A**,**C**) and W1PR2 (**B**,**D**) cells cultured under monolayer conditions following treatment with LDE225 or Vismodegib. Protein levels were assessed via Western blot analysis. GAPDH served as the loading control. Densitometric measurements are presented in charts below the corresponding blots. Statistical significance was assessed using Student’s *t*-test, with * *p* < 0.05 considered statistically significant.

Afterwards, we checked whether the tested SMO inhibitors would change the expression of ALDH1A1, a universal marker of CSCs. As the W1 and W1PR1-C7 cell lines do not express this protein, the effect of LDE225 and VIS on ALDH1A1 expression was investigated only in W1PR1 and W1PR2 cell lines. In the W1PR1 cell line, we noted the same level of ALDH1A1 protein expression regardless of LDE225 or VIS treatment (Figure 11A,C). In contrast, some decrease in ALDH1A1 protein level after both inhibitors’ treatment was observed in the W1PR2 cell line (*p* < 0.05) (Figure 11B,D).

#### 2.5.3. Effect of SMO Inhibitors on COL3A1 Protein Expression

**Figure 12:**
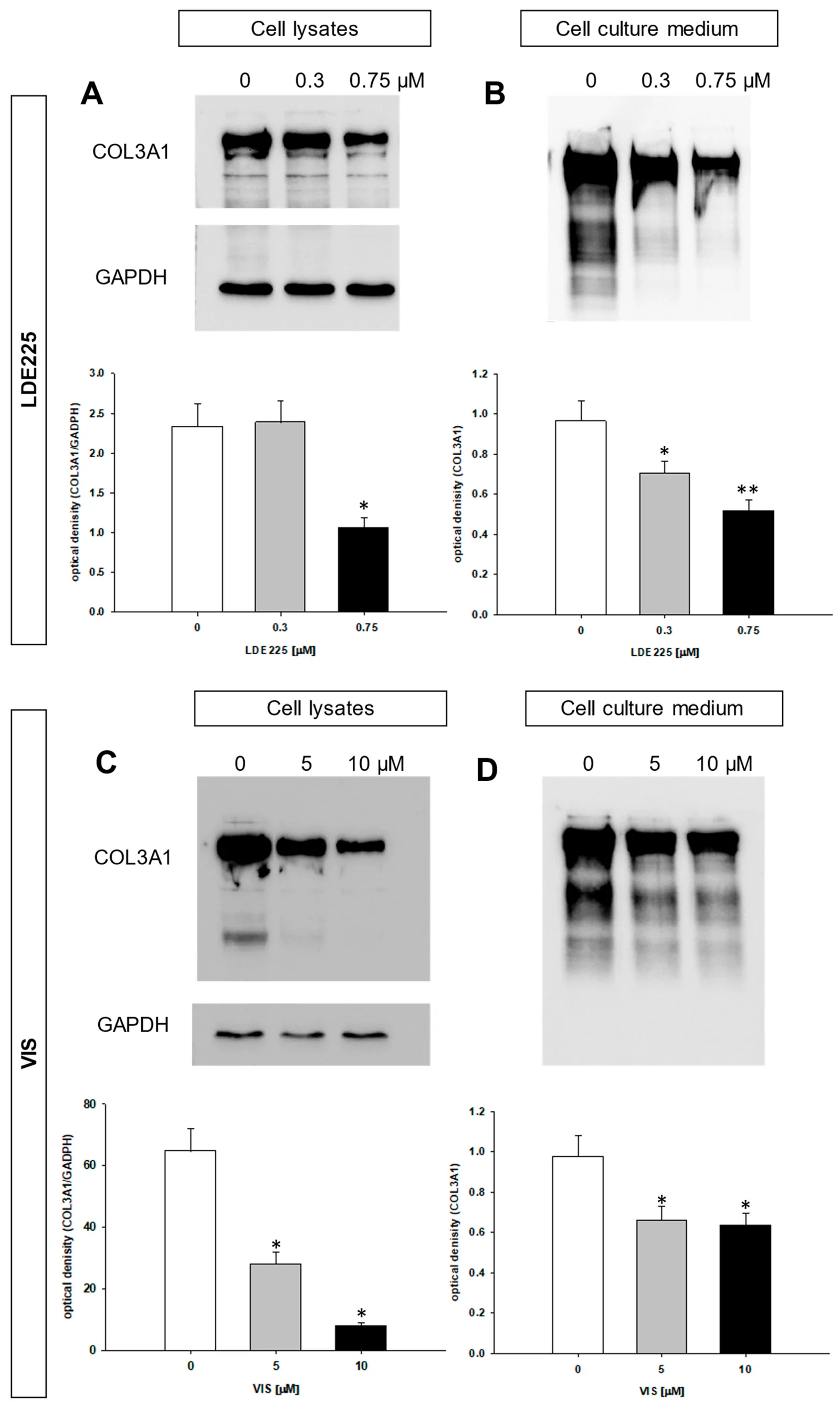
Effect of LDE225 (**A**,**B**) and Vismodegib (**C**,**D**) on COL3A1 protein expression in W1PR2 cells. COL3A1 protein expression was analyzed in cell lysates (**A**,**C**) and conditioned culture medium (**B**,**D**) from W1PR2 cells grown under monolayer conditions following treatment with LDE225 or Vismodegib. Protein levels were assessed via Western blot analysis. GAPDH served as the loading control for cell lysates. Densitometric measurements are presented in charts below the corresponding blots. Statistical significance was assessed using Student’s *t*-test, with * *p* < 0.05 and ** *p* < 0.01 considered statistically significant.

In the W1PR2 cell line, we noted strong overexpression of COL3A1 protein. Thus, we wanted to establish whether treatment with LDE225 or VIS would impact the expression of this protein. As COL3A1 is a secreted protein, the expression was investigated in both cell lysates and cell culture medium. Treatment with LDE225 resulted in a downregulation of a band with a higher molecular mass in material derived from cell lysates, with a statistically significant decrease in concentration of 0.75 µM (*p* < 0.05) (Figure 12A). A stronger concentration-dependent effect of LDE225 was observed in the cell culture medium. We noted a reduction in the expression of COL3A1, especially a strong reduction in the bands with lower molecular mass, but also a clear decrease in expression of bands with higher molecular mass (*p* < 0.05, *p* < 0.01) (Figure 12B). VIS treatment caused a clear reduction of COL3A1 expression in both cell lysates (*p* < 0.05) (Figure 12C) and cell culture medium (*p* < 0.05) (Figure 12D).

**Figure 13:**
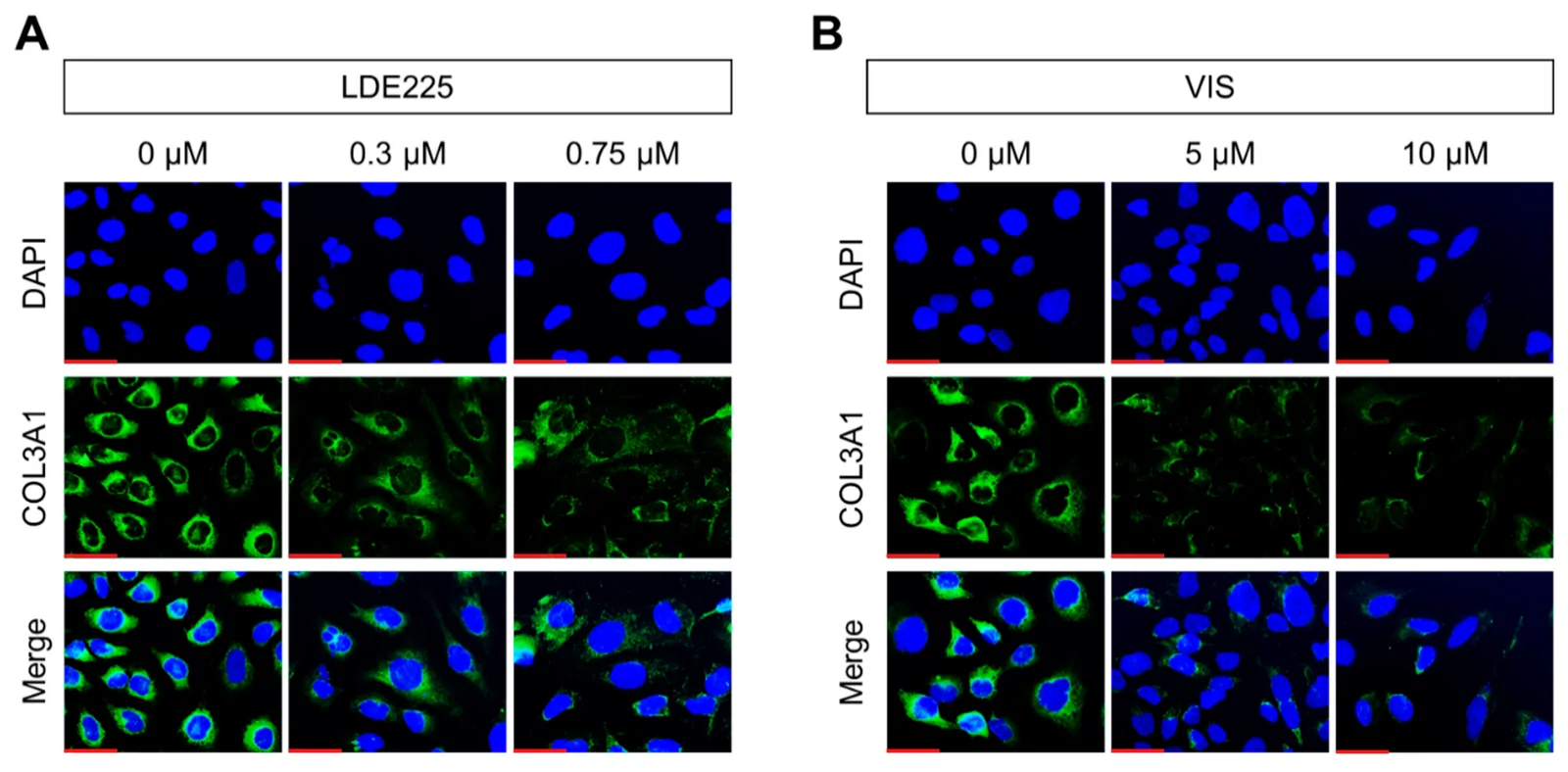
COL3A1 immunofluorescence in W1PR2 cells following LDE225 (**A**) or Vismodegib (**B**) treatment. COL3A1 was detected using an anti-COL3A1 antibody followed by Alexa Fluor^®^488-conjugated secondary antibody (green). Nuclei were counterstained with DAPI (blue). Images were acquired at 40× magnification. Scale bar = 50 μm.

To confirm the effect of both inhibitors on COL3A1 protein expression, we performed immunofluorescence analysis as well. We noted no effect on fluorescence intensity after LDE225 treatment (Figure 13A). In contrast, a clear decrease in fluorescence intensity was visible following treatment with VIS at concentrations of 5 and 10 µM (Figure 13B).

### 2.6. The Effect of LDE225 on Cell Proliferation

**Figure 14:**
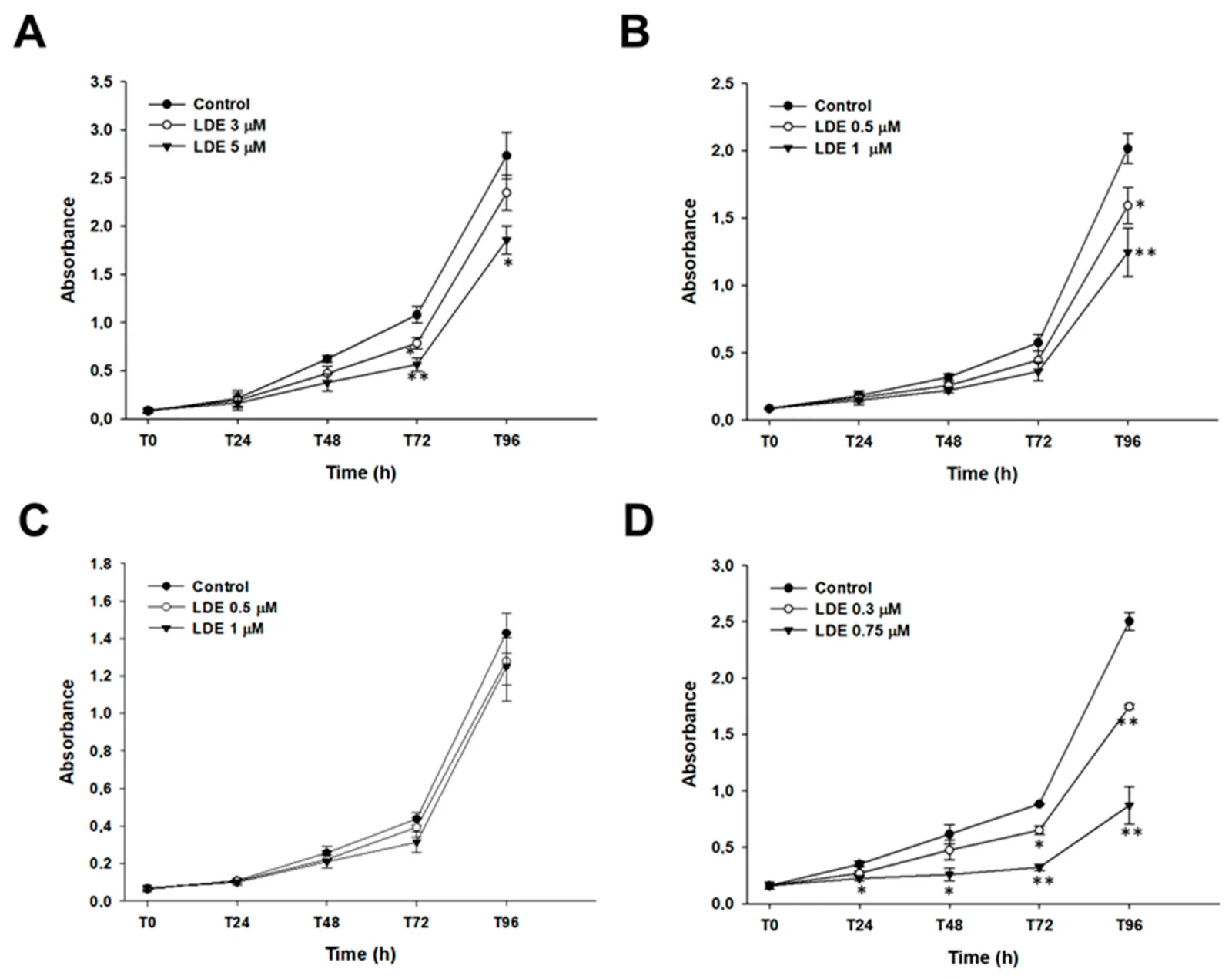
Effect of LDE225 on cell proliferation. Proliferation of W1 (**A**) and paclitaxel-resistant cell lines W1PR1 (**B**), W1PR1-C7 (**C**), and W1PR2 (**D**) was assessed following LDE225 treatment. Cell viability was measured every 24 h using the MTT assay. Statistical significance is indicated as * *p* < 0.05, and ** *p* < 0.01.

**Figure 15:**
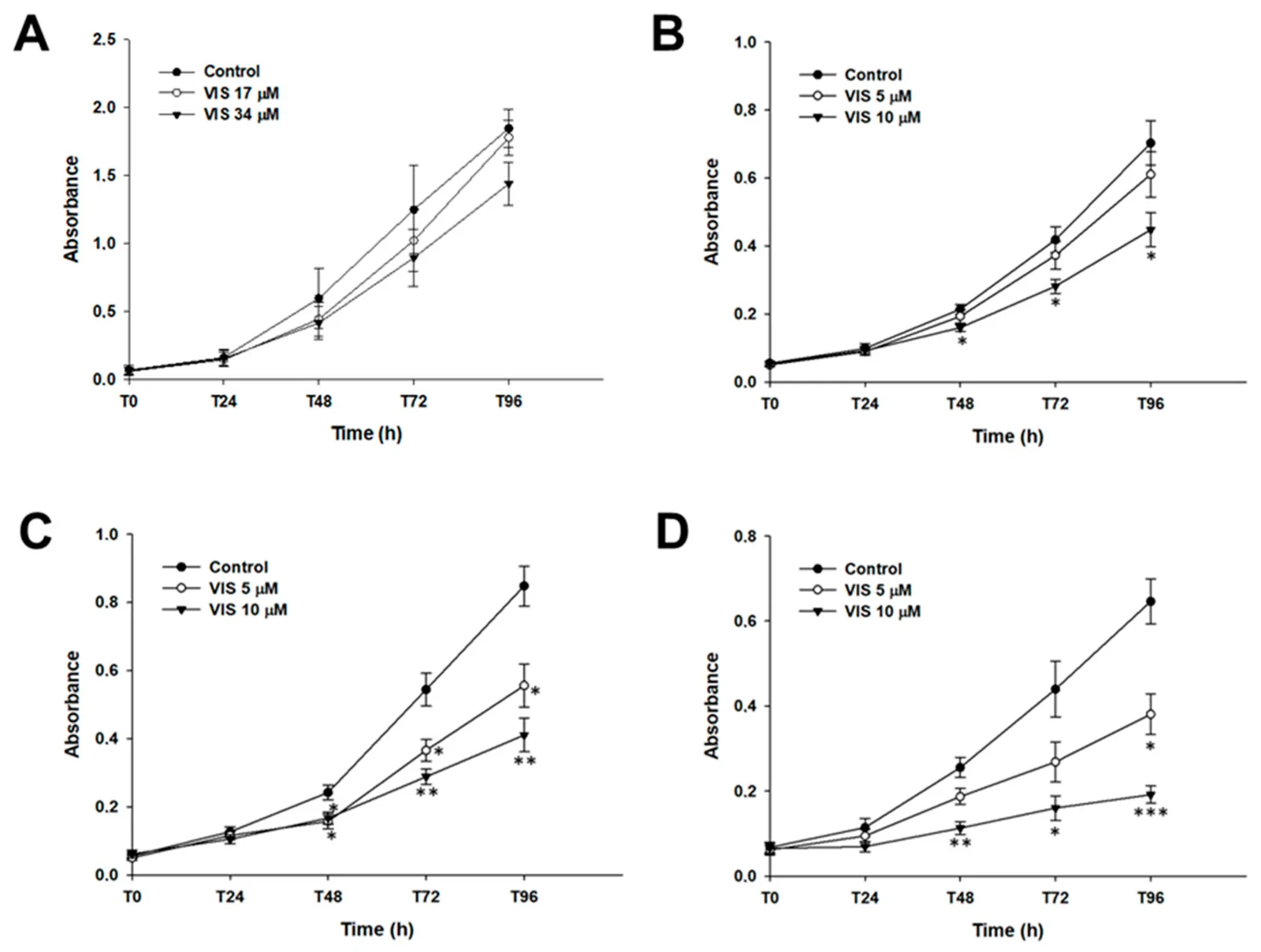
Effect of Vismodegib on cell proliferation. Proliferation of W1 (**A**) and paclitaxel-resistant cell lines W1PR1 (**B**), W1PR1-C7 (**C**), and W1PR2 (**D**) was assessed following Vismodegib treatment. Cell viability was measured every 24 h using the MTT assay. Statistical significance is indicated as * *p* < 0.05, ** *p* < 0.01, and *** *p* < 0.001.

The effect of LDE225 on the proliferation of ovarian cancer cells was also investigated (Figure 14). IC_50_ concentration reduced cell proliferation in the W1 cell line after 72 h (*p* < 0.01) and 96 h (*p* < 0.05) of treatment (Figure 14A) and in the W1PR1 cell line after 96 h of treatment (*p* < 0.01) (Figure 14B). No effect of LDE225 at the IC_50_ concentration was observed in the W1PR1-C7 cell line (Figure 14C). In contrast, the proliferation of the W1PR2 cell line was reduced at all time points (*p* < 0.05, *p* < 0.01, *p* < 0.001) (Figure 15D). IC_25_ concentration reduced cell proliferation after 72 h in the W1 cell line (*p* < 0.05) (Figure 14A), after 96 h in the W1PR1 cell line (*p* < 0.05) (Figure 14B), and after 72 h (*p* < 0.05) and 96 h (*p* < 0.01) in the W1PR2 cell line (Figure 14D).

### 2.7. The Effect of VIS on Cell Proliferation

Next, we investigated the effect of VIS on the proliferation of the cell lines (Figure 15). No statistically significant effect of VIS at concentrations of IC_25_ and IC_50_ on the proliferation of the W1 cell line was observed (Figure 15A). In the W1PR1 cell line, we observed a reduction in proliferation rate at the IC_50_ VIS concentration at 48 h, 72 h, and 96 h (*p* < 0.05) (Figure 15B). A similar effect was observed in the W1PR1-C7 (Figure 15C) and the W1PR2 (Figure 15D) cell lines (*p* < 0.05, *p* < 0.01, or *p* < 0.001). IC_25_ concentration reduced cell proliferation after 72 h and 96 h of treatment (*p* < 0.05) in the W1PR1-C7 cell line (Figure 15C) and after 96 h in the W1PR2 cell line (*p* < 0.05) (Figure 15D).

### 2.8. The Effect of SMO Inhibitors on Colony Formation

**Figure 16:**
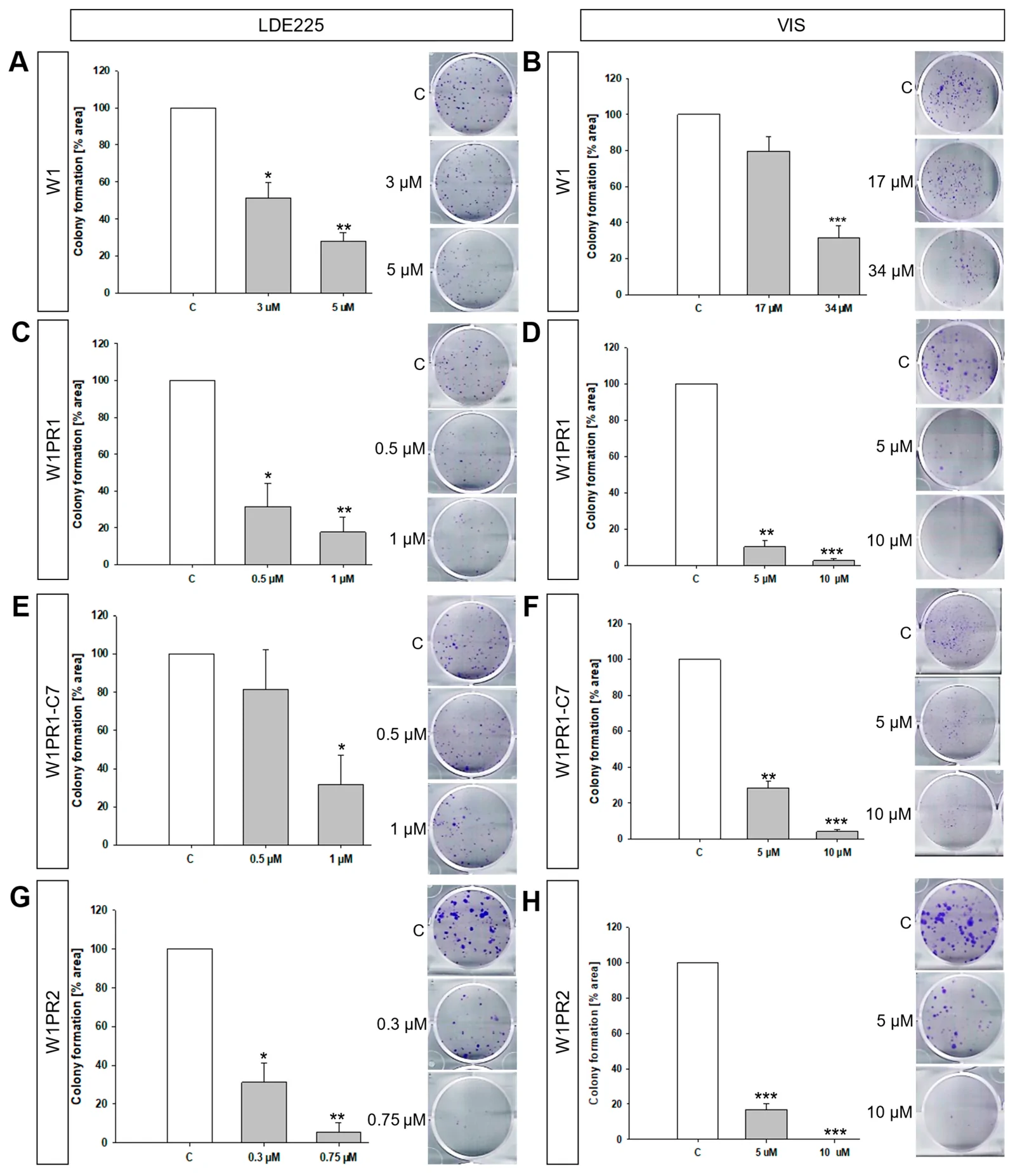
Colony formation assay following treatment with SMO inhibitors (mean from at least 3 independent experiments). Colony formation in W1 (**A**,**B**), W1PR1 (**C**,**D**), W1PR1-C7 (**E**,**F**), and W1PR2 (**G**,**H**) cells was assessed after treatment with LDE225 (**A**,**C**,**E**,**G**) or Vismodegib (**B**,**D**,**F**,**H**). Cells were cultured for 7 days with the indicated inhibitors, then fixed and stained using crystal violet. Colony formation efficiency was quantified as the percentage of the area of colonies formed. Statistical significance was assessed using Student’s *t*-test. Statistical significance is indicated as * *p* < 0.05, ** *p* < 0.01, and *** *p* < 0.001.

To further investigate the effect of inhibitors on ovarian cancer cells, we checked the effect on colony formation using concentrations corresponding to IC_25_ and IC_50_. The effect of LDE225 on colony formation is presented in Figure 16A,C,E,G. Treatment with LDE225 resulted in a decrease in the area of colonies formed in all of the tested cell lines, and the effect was concentration-dependent. Treatment with LDE225 at a concentration equal to IC_25_ resulted in a statistically significant decrease in colony area in W1 (50% of control), W1PR1, and W1PR2 (30% of control) cell lines, *p* < 0.05, but not in the W1PR1-C7 cell line. Treatment with IC_50_ concentration resulted in a further decrease in colony area: W1 (30% of control, *p* < 0.01), W1PR1 (10% of control, *p* < 0.01), W1PR1-C7 (30% of control, *p* < 0.05), and W1PR2 (5% of control, *p* < 0.01).

Next, the same cell lines were treated with VIS at concentrations of IC_25_ and IC_50_ (Figure 16B,D,F,H). In the W1 cell line, we did not observe a statistically significant effect of VIS at a concentration of IC_25_. Treatment with VIS at an IC_50_ concentration resulted in a reduction of colony area to 30% of the control, *p* < 0.001. Treatment of PAC-resistant cell lines with IC_25_ concentration resulted in a strong reduction of colony-forming area to 10% of control in the W1PR1 cell line, *p* < 0.01, 30% of control in the W1PR1-C7 cell line, *p* < 0.01, and 20% of control in the W1PR2 cell line, *p* < 0.001. VIS at the IC_50_ concentration resulted in a very strong reduction in colony-forming area to about 3% of control in the W1PR1 cell line, *p* < 0.001; 5% of control in the W1PR1-C7 cell line, *p* < 0.001, and nearly zero in the W1PR2 cell line, *p* < 0.001.

### 2.9. Comparison of Protein Expression in 2D and 3D Cell Culture Model

**Figure 17:**
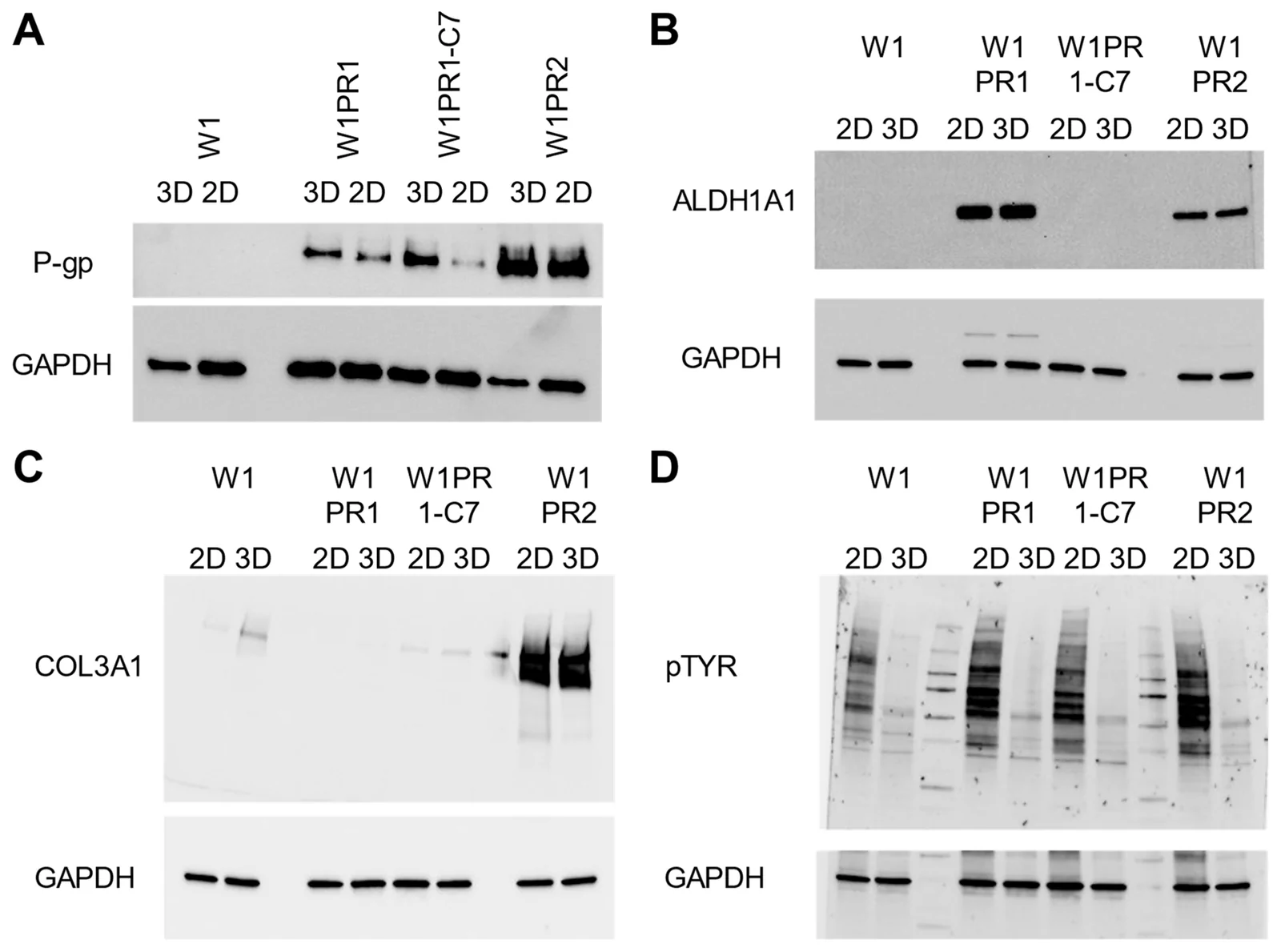
Comparison of protein expression in 2D and 3D culture conditions. Western blot analysis of P-gp (**A**), ALDH1A1 (**B**), COL3A1 (**C**), and pTYR (**D**) in cell lines cultured under monolayer (2D) and spheroid (3D) conditions. Protein levels were assessed via Western blot analysis. GAPDH served as the loading control.

**Figure 18:**
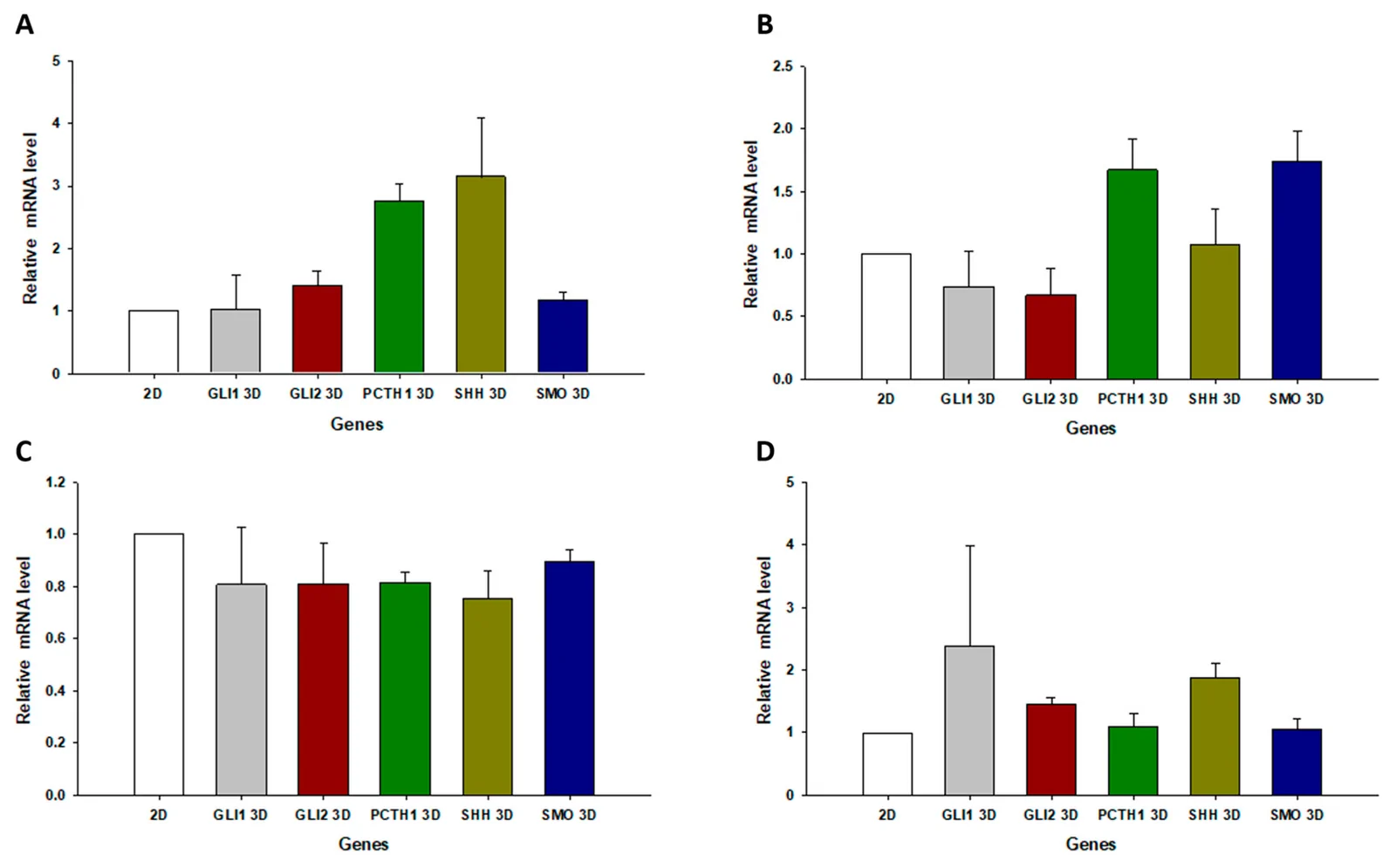
Analysis of gene expression in W1 (**A**) and paclitaxel-resistant sublines W1PR1 (**B**), W1PR1-C7 (**C**), and W1PR2 (**D**) under 2D and 3D culture conditions. Expression of GLI1, GLI2, GLI3, PTCH1, SHH, and SMO was assessed via qPCR. Results are presented as relative expression in 3D cultures normalized to the corresponding 2D cultures of the same cell line (set to 1).

In the next part of our study, we wanted to assess the effects of both SMO inhibitors on drug resistance in a 3D model. First, we compared the expression level of drug-resistant proteins in cell lines growing in 2D and 3D cell culture conditions. We observed a very similar pattern of P-gp (Figure 17A), ALDH1A1 (Figure 17B), and COL3A1 (Figure 17C) expression in the same cell lines growing in 2D and 3D cell culture conditions. We also compared Shh pathway gene expression in the same cell line growing in 2D and 3D cell culture conditions, although we did not observe any significant changes in gene expression (Figure 18). In all cell lines, we also observed a strong decrease in pTYR in 3D spheroids in comparison to 2D monolayers (Figure 17D).

### 2.10. The Response of Drug-Sensitive and Resistant Cell Lines to LDE225 Treatment in a 3D Model

**Figure 19:**
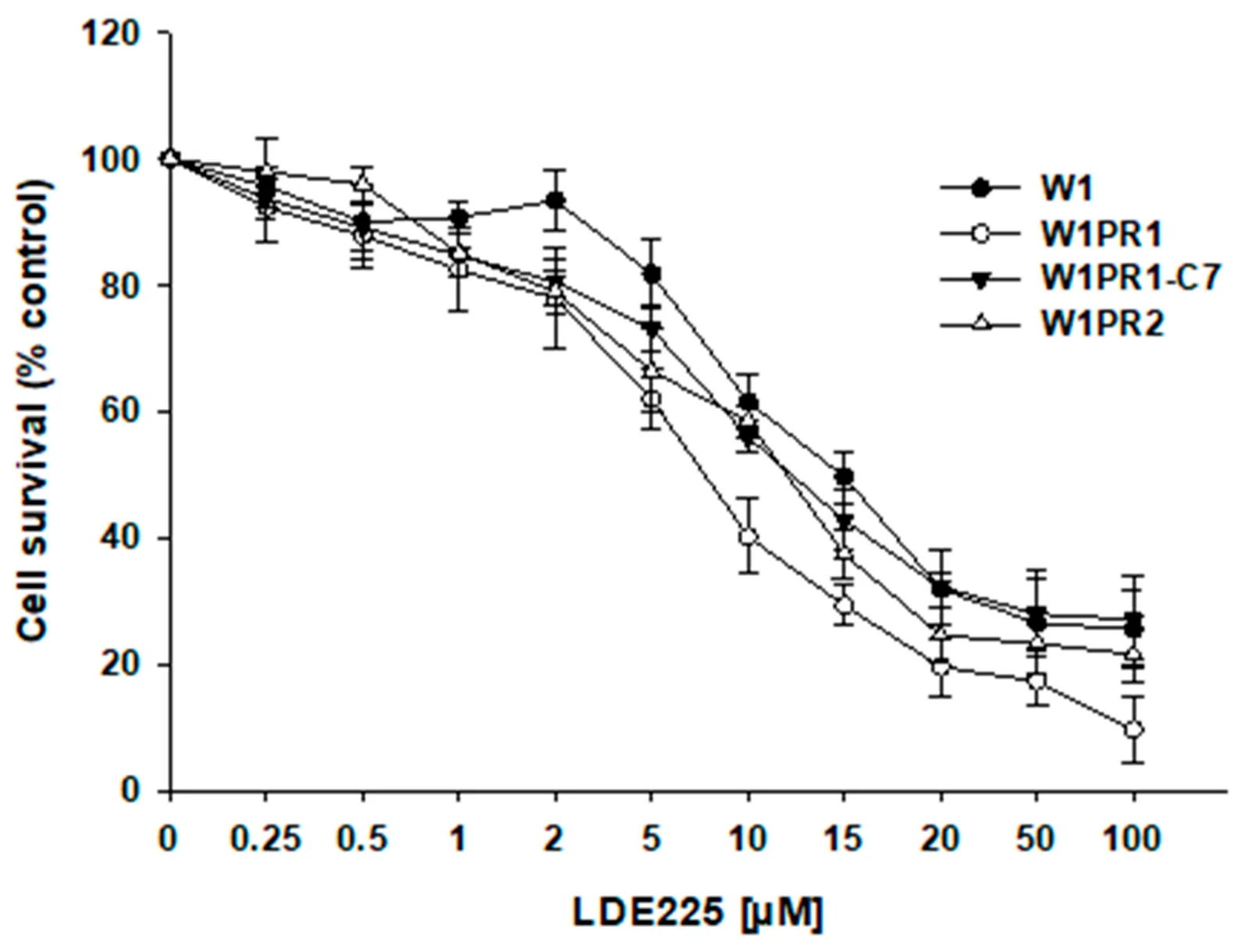
Effect of LDE225 on cell viability in 3D spheroid cultures. W1 and paclitaxel-resistant sublines (W1PR1, W1PR1-C7, and W1PR2) were cultured under 3D conditions and treated with increasing concentrations of LDE225 for 72 h. Cell viability was determined using the MTT assay, and results are presented as mean ± SEM and normalized to the untreated control.

We observed similar response curves to LDE225 treatment in all investigated cell lines. The most resistant cell line was the W1 cell line, and the most sensitive was the W1PR2 cell line (Figure 19). Based on the response curves, the IC_50_ value of LDE225 was calculated (Table 5). The IC_50_ values were 8.06 µM in the W1PR1 cell line vs. 14.32 µM in the W1 cell line and were decreased 1.78-fold (*p* < 0.05); 12.9 µM in the W1PR1-C7 cell line vs. 14.32 µM in the W1 cell line and were decreased 1.11-fold (*p* not statistically significant); 12.15 µM in the W1PR2 cell line vs. 14.32 µM in the W1 cell line and were decreased 1.18-fold.

### 2.11. The Response of Drug-Sensitive and Resistant Cell Lines to VIS Treatment in the 3D Model

**Figure 20:**
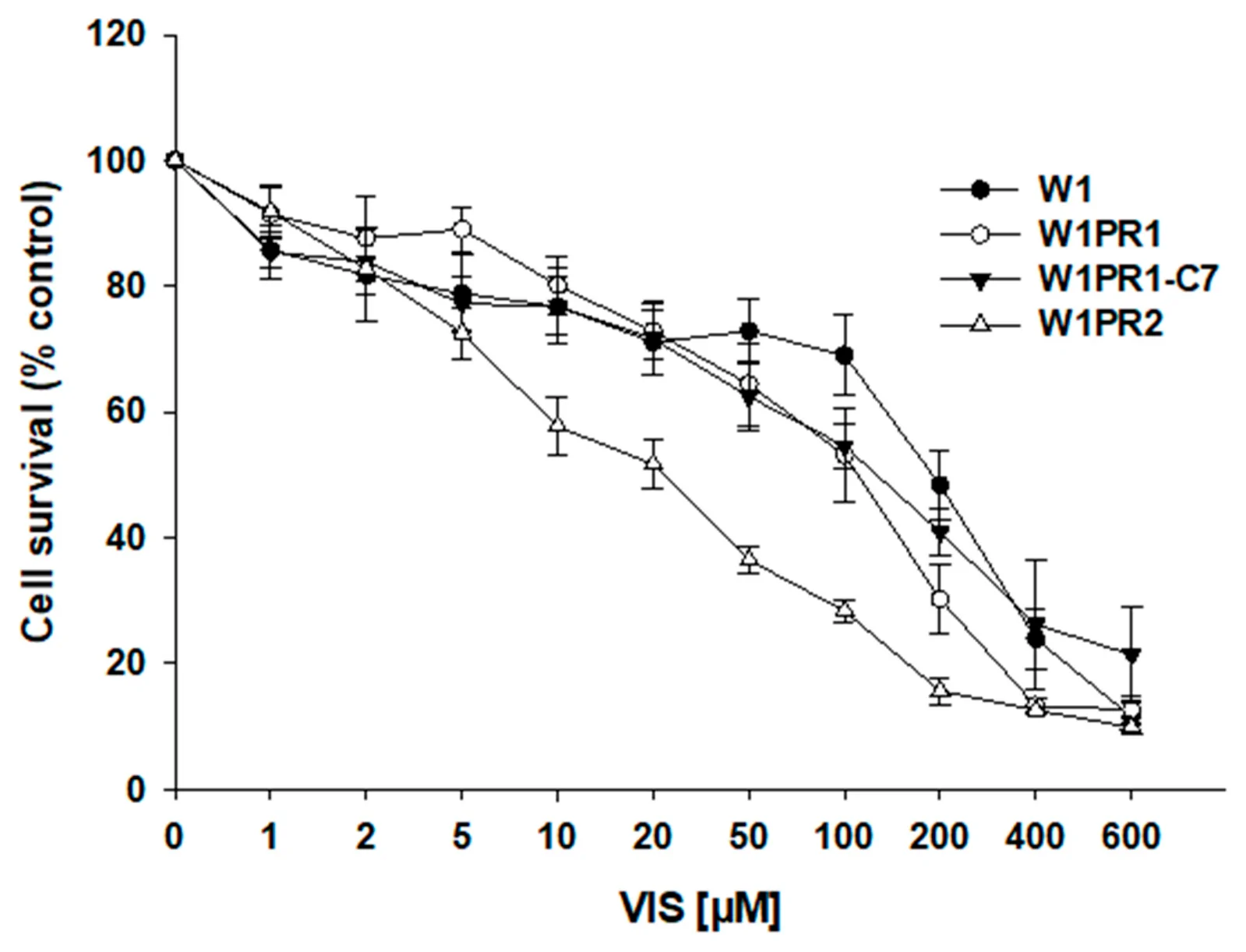
Effect of VIS on drug-sensitive cell line and paclitaxel-resistant sublines under 3D culture conditions. All cell lines were seeded at 1 × 10^4^ cells per sphere and were cultured for 72 h without or with increased concentrations of VIS. Results are presented as mean ± SEM and normalized to the untreated control.

We also investigated the effect of VIS on cell viability in the 3D model. In the W1 cell line, we observed a mild concentration-dependent effect of VIS at concentrations up to 100 µM. A higher increase in VIS concentration resulted in a more rapid concentration-dependent decrease in cell viability. PAC-resistant cell lines showed a more uniform concentration-dependent response, with a similar level of resistance in the W1PR1 and the W1PR1-C7 cell lines. In contrast, the W1PR2 was more resistant to VIS treatment (Figure 20). Based on the cellular response, we calculated IC_50_ values (Table 6). In PAC-resistant cell lines, the IC_50_ value was lower than in the W1 cell line. In detail, in the W1PR1 cell line, the IC_50_ value was 110 µM vs. 183 µM in the W1 cell line and it decreased 1.67-fold (*p* not statistically significant); in the W1PR1-C7 cell line, the IC_50_ value was 130 µM vs. 183 µM in the W1 cell line and it decreased 1.41-fold (*p* not statistically significant); in the W1PR2 cell line, the IC_50_ value was 25.1 µM vs. 188 µM in the W1 cell line and it decreased 7.33-fold (*p* < 0.01).

### 2.12. Comparison of the Effect of LDE and VIS in 2D and 3D Models

**Figure 21:**
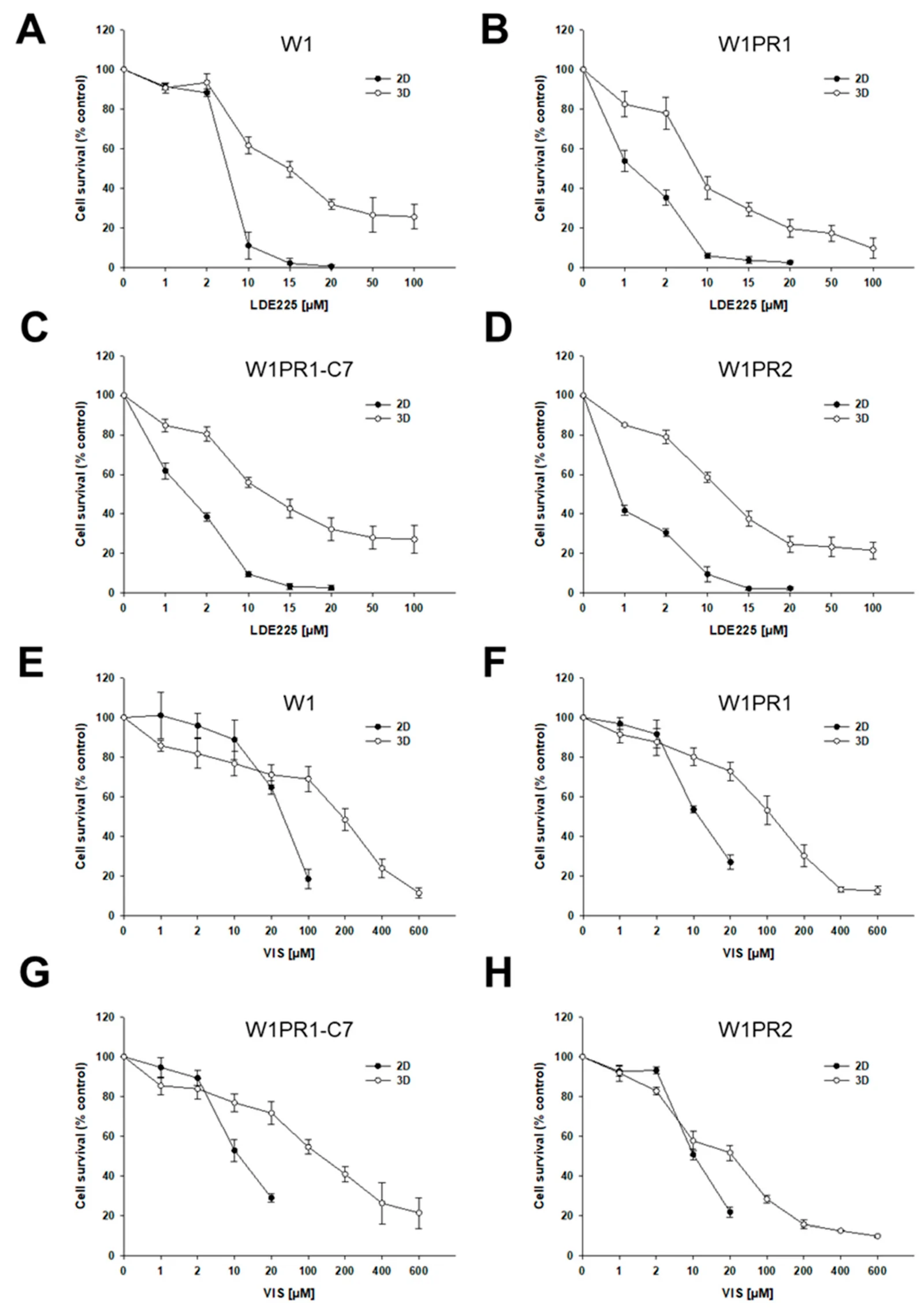
Comparison of the effect of SMO inhibitors in 2D and 3D models. Sensitive cell line (**A**,**E**) and paclitaxel-resistant sublines: W1PR1 (**B**,**F**), W1PR1-C7 (**C**,**G**), and W1PR2 (**D**,**H**) were seeded into 96-well plates as monolayers or 3D spheroids and were treated with LDE225 (**A**–**D**) or VIS (**E**,**F**). Following a 72-h incubation, the cell survival assay (MTT) was performed. Results are presented as mean ± SEM and normalized to the untreated control.

Next, we compared the effect of LDE in the same cell lines cultured as 2D monolayers and 3D spheroids (Figure 21A–D, Table 7). In the W1 cell line grown as 3D spheroids, we observed a 2.85-fold increase in IC_50_ value in comparison to the 2D monolayer (IC_50_ = 14.32 µM vs. IC_50_ = 5.03 µM, *p* < 0.01). In the W1PR1 cell line, we observed a 6.34-fold higher IC_50_ value in spheroids than in the monolayer (IC_50_ = 8.06 µM vs. IC_50_ = 1.27 µM, *p* < 0.05). The W1PR1-C7 cell line was characterized by an 8.85-fold higher IC_50_ value in spheroids than in the monolayer (IC_50_ = 12.9 µM vs. IC_50_ = 1.45 µM, *p* < 0.01). The highest difference was observed in the W1PR2 cell line, where we observed a 16-fold increase in IC_50_ values (IC_50_ = 12.15 µM vs. IC_50_ = 0.76 µM, *p* < 0.001).

Similarly, we compared the effect of VIS (Figure 21E,F) in 2D and 3D cell culture conditions. In the W1 cell line growing as 3D spheroids, we observed a 5.42-fold increase in IC_50_ value in comparison to the 2D monolayer (IC_50_ = 183 µM vs. IC_50_ = 33.9 µM, *p* < 0.01). In the W1PR1 cell line, we observed a 9.89-fold higher IC_50_ value in spheroids than in the monolayer (IC_50_ = 110 µM vs. IC_50_ = 11.1 µM, *p* < 0.01). In the W1PR1-C7 cell line, we observed an 11.7-fold higher IC_50_ value in spheroids than in the monolayer (IC_50_ = 130 µM vs. IC_50_ = 11.1 µM, *p* < 0.05). W1PR2 cell line was characterized by a 2.43-fold increase in IC_50_ value (IC_50_ = 25.1 µM vs. IC_50_ = 10.3 µM, *p* < 0.01).

### 2.13. The Effect of LDE and VIS Treatment on the Spheroid Structure and Viability

**Figure 22:**
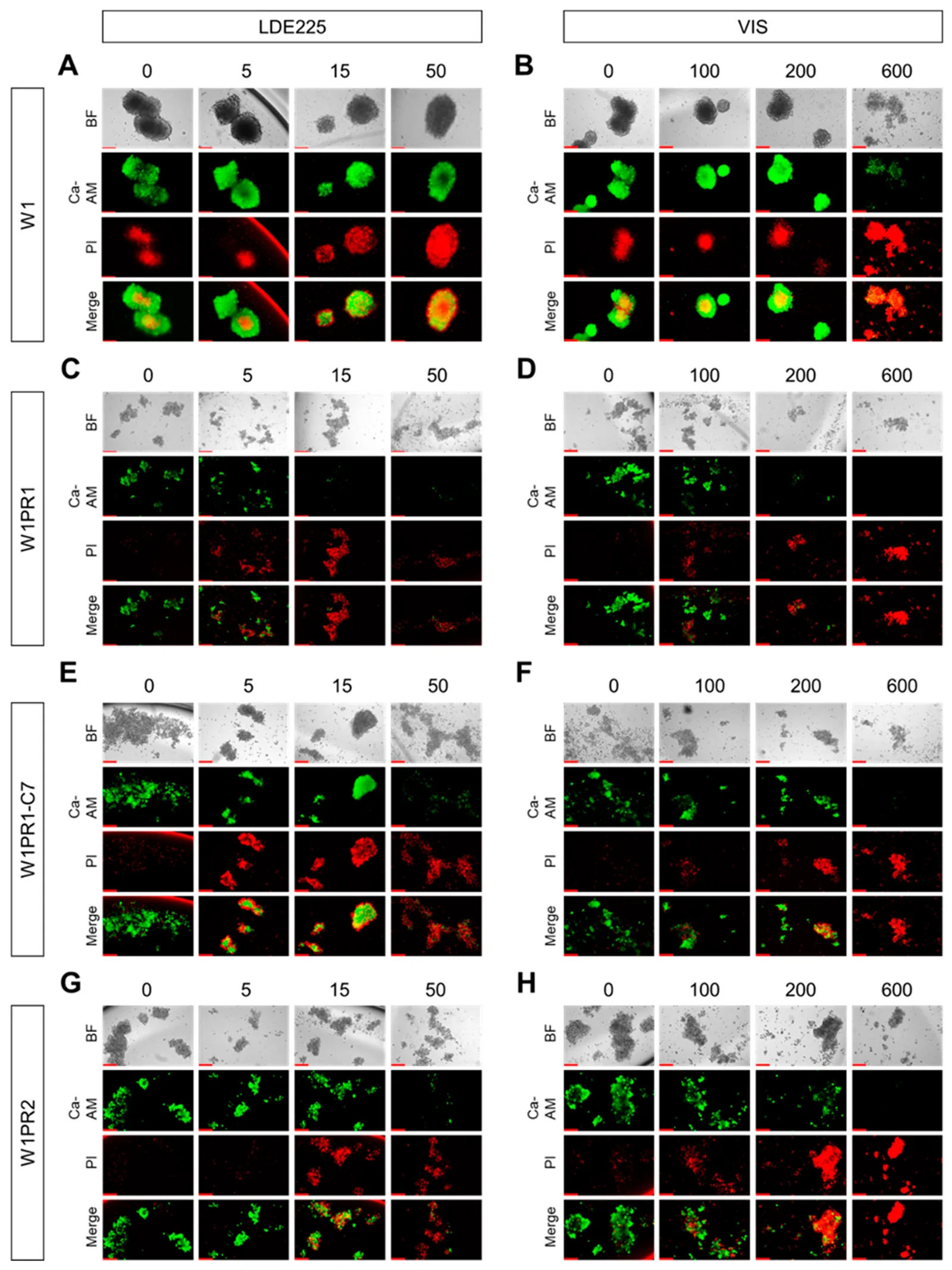
Fluorescence microscopy analysis of Calcein-AM (green) and PI (red) accumulation in a 3D model. The paclitaxel-sensitive W1 cell line (**A**,**B**) and the resistant sublines W1PR1 (**C**,**D**), W1PR1-C7 (**E**,**F**), and W1PR2 (**G**,**H**) were cultured with or without LDE225 (**A**,**C**,**E**,**G**), at concentrations of 5, 15, and 50 µM. The same cell lines were treated with VIS (**B**,**D**,**F**,**H**), at concentrations of 0, 100, 200, and 600 µM. Cells were treated with LDE225 or VIS for 72 h, and next, a fluorescence analysis was performed. Scale bar: 200 μm.

Next, we investigated the structure and viability of spheroids after 72 h of treatment with LDE225 or VIS. First, two types of spheroids were observed in the investigated cell lines. W1 cell lines formed very dense circular spheroids, while W1PR1 and W1PR1-C7 cell lines formed low-density spheroids. W1PR2 cell line formed spheroids with medium density (Figure 22). After labelling W1-derived spheroids with Calcein-AM and PI, we observed a necrotic core labelled by PI and less by Calcein-AM. Some necrotic cells were also present in spheroids from other cell lines. After treatment with increased concentrations of LDE225 in the W1 cell line (Figure 22A), we observed loosening of spheroid structure and increased PI signal, especially at concentrations of 15 and 50 µM. An increase in LDE225 concentration also resulted in decreased CA-AM signal, especially at a concentration of 50 µM. In PAC-resistant cell lines, we observed a significant increase in PI accumulation following LDE225 treatment. The accumulation was clearly visible, starting at a concentration of 5 µM in W1PR1 and W1PR1-C7 cell lines (Figure 22C,E) and 15 µM in the W1PR2 cell line (Figure 22G). In contrast, LDE225 treatment resulted in a concentration-dependent decrease in CA-AM signal from 15 µM in the W1PR1 cell line and from 50 µM in the W1PR1-C7 and W1PR2 cell lines.

Treatment of the W1 cell line with increasing VIS (Figure 22B) concentrations resulted in loosening of spheroid density, clearly visible at a concentration of 600 µM. This was associated with a clear increase in PI labelling and a decrease in CA-AM labelling. A very similar response pattern to LDE225 was also observed for VIS in the PAC-resistant cell lines. We observed a concentration-dependent increase in PI staining and a concentration-dependent decrease in staining with CA-AM. A total lack of CA-AM staining was observed at a concentration of 600 µM.

## 3. Discussion

The development of drug resistance is a key mechanism underlying chemotherapy failure in ovarian cancer [1,2]. Both mechanisms related to individual cancer cells [4,5,10,11] and tumor tissue organization [13,14,15,21,22] play a role in this phenomenon. According to the CSCs model of drug resistance, CSCs are responsible for drug resistance in cancers [24]. As the biology of CSCs is regulated by the CSCs signaling pathways, these pathways are attractive therapeutic targets. Among them, the Sonic Hedgehog pathway is involved in ovarian cancer development [40,41].

In this study, we compared the effects of LDE225 and Vismodegib, two SMO protein antagonists approved for cancer treatment [38,47], in PAC-sensitive and PAC-resistant ovarian cancer cell lines [51,53].

We first characterized the investigated cell lines. As ALDH1A1 is the most common marker of CSCs in solid tumors [24], we checked its expression. Both PAC-resistant cell lines showed increased expression of ALDH1A1 at transcript and protein levels and the presence of both ALDH1A1^+^ and ALDH1A1^−^ cell populations, as previously reported by others in PAC-resistant ovarian cancer cell lines [29,31,32]. In contrast, we did not observe ALDH1A1 expression in the W1PR1-C7 cell line, with the knockdown of this gene, consistent with our previous study [51]. As PAC resistance is commonly associated with P-gp overexpression [4,5], we examined its expression in PAC-resistant and sensitive cell lines. All PAC-resistant cell lines showed P-gp overexpression and membrane localization, indicating its role in PAC resistance. Drug resistance can also be related to overexpression of ECM molecules, which we previously observed in our drug-resistant cell lines [16,17,18]. Here, we confirmed strong COL3A1 overexpression in W1PR2, suggesting its role in PAC resistance. As increased protein phosphorylation is often observed in drug-resistant cells [10,11], we also compared pTYR levels and observed an increase in all PAC-resistant cell lines, consistent with our previous study [12]. Summarizing, our PAC-resistant cell lines presented features of CSCs and a drug-resistance mechanisms typical of drug-resistant cells.

Next, we compared the expression of Shh pathway genes among the cell lines. The expression of genes encoding *PTCH1*, *SHH*, and *SMO* was similar in drug-sensitive and resistant cell lines, suggesting comparable activity of this pathway, as previously observed in TOP-resistant A2780 ovarian cancer cell lines [54]. In contrast, *GLI* gene expression differed between cell lines, with *GLI1* downregulated and *GLI2* upregulated in W1PR1 and W1PR1-C7 cell lines, while both genes remained unchanged in W1PR2 compared to the drug-sensitive control. Similarly, a study comparing three pairs of drug-sensitive and resistant ovarian cancer cell lines found no consistent pattern of changes in *SHH*, *PTCH1*, *SMO*, *GLI1*, and *GLI2* expression, with some genes upregulated in a cell line-dependent manner [36].

After assessing Shh pathway gene expression, we compared the response of the investigated cell lines to two SMO inhibitors, LDE225 and VIS. All PAC-resistant cell lines showed a few-fold lower IC_50_ values for LDE225 (0.76–1.45 µM) than the drug-sensitive W1 cell line (5.03 µM), suggesting a potential association between Shh pathway-related signaling and LDE225 sensitivity. Similarly, in another study, drug-sensitive ovarian cancer cell lines showed approximately two-fold higher sensitivity to LDE225 than their drug-resistant counterparts, although the reported IC_50_ values were higher (12–24 µM and 7.5–12 µM, respectively) [36]. IC_50_ values in the µM range were also reported for human renal cell carcinoma cell lines (1–5 µM) [45], and human melanoma cell lines (5–10 µM) [55]. Thus, our results are consistent with previous studies, and the higher sensitivity of PAC-resistant cells suggests that LDE225 may be a promising therapeutic option for drug-resistant ovarian cancer.

A similar sensitivity pattern was observed for VIS, with a higher IC_50_ in the drug-sensitive cell line (34 µM) than in PAC-resistant cell lines (10.3–11.1 µM). Although no studies comparing VIS sensitivity in drug-sensitive and resistant pairs of cell lines were found, IC_50_ values in the µM range have been reported for cell lines derived from lung cancer (50 µM) [56], neuroblastoma (61–77 µM) [57], medulloblastoma (52–84 µM) [58], and pancreatic cancer (5–10 µM) [59], consistent with our findings.

Comparison of IC_50_ values for LDE225 and VIS indicates that the IC_50_ for VIS was always 5–10-fold higher than for LDE225 in the same cell line, indicating a higher potency of LDE225 as an inhibitor of the Shh pathway in our model. In general, the IC_50_ values are usually lower for LDE225 than for VIS, although this is not a universal rule, as observed in breast cancer cell lines [60].

Summarizing this part of the study, our IC_50_ values for both LDE225 and VIS were relatively low and further decreased in PAC-resistant cell lines, indicating that both SMO inhibitors may be potential candidates for ovarian cancer treatment.

Stage et al. reported that inhibition of SMO by LDE225 and cyclopamine, a naturally occurring SMO inhibitor that inspired the development of VIS, increased PAC sensitivity in drug-resistant ovarian cancer cell lines through *MDR1* downregulation [36]. In another study, the treatment of DM14 (metastatic squamous cell carcinoma), LNCaP (prostate carcinoma), PC3 (prostate carcinoma), SEG-1 (esophageal adenocarcinoma) cell lines with cyclopamine resulted in downregulation of P-gp and BCRP protein expression and increased accumulation of cytotoxic drugs in the cells [61]. The role of the Shh pathway in the regulation of P-gp and BCRP was also suggested in ovarian cancer [62], colorectal cancer [63], and oral squamous cell carcinoma [64]. Thus, we investigated whether SMO inhibitors would have a similar effect in our cell lines. However, treatment with PAC supplemented with LDE225 or VIS did not result in increased PAC sensitivity, and no statistically significant differences in IC_50_ values for PAC were observed. Similarly, we did not observe changes in P-gp protein expression after treatment with either inhibitor, except for its downregulation in the W1PR1-C7 cells treated with LDE225.

Others reported downregulation of *ALDH* expression after VIS treatment in colon cancer cell lines [65] and a decrease in the number of ALDH+ cells in pancreatic cancer cell lines after cyclopamine treatment [66]. In contrast to these studies, we did not observe changes in the expression of ALDH1A1, a marker of CSCs, after either SMO inhibitor treatment. These results suggest that P-gp expression and CSC biology are not regulated by the Shh pathway in the investigated cell lines.

In contrast, we observed a downregulation of COL3A1 protein, especially a strong decrease in the protein secreted into the cell culture medium, after treatment with both inhibitors. We did not find any reports describing the relationship between SMO inhibitor treatment and COL3A1 expression. According to the ECM model of drug resistance development, cancer cells overexpressing extracellular matrix molecules are more resistant to chemotherapy [13,15]. Recently, in the W1PR2 cell line, we observed abundant overexpression of COL3A1 protein in cell lysates and medium [67], and cells expressing higher COL3A1 levels were ALDH1A1+ [17,67]. ECM molecules, such as COL3A1, can influence drug resistance in several ways: by inducing CAM-DR, resulting in increased signal transduction, expression of drug-resistance genes, and higher resistance to apoptosis [13,15]; by binding some cytotoxic drugs, such as DOX, MTX, or PAC, limiting their accessibility to cancer cells [14]; and by limiting drug diffusion, especially in drug-resistant tumors, resulting in delayed penetration and lower drug concentrations in the tumor [21,68]. Thus, the observed downregulation of COL3A1 after LDE225 and VIS treatment should result in decreased activity of this drug resistance mechanism.

Next, we investigated the effect of both inhibitors on the replication potential of cancer cells, using proliferation and colony formation assays.

Treatment of the investigated cell lines with LDE225 or VIS generally resulted in reduced cell proliferation, although the effect was cell line-dependent and not always observed. In a similar study, in A2780 and TOP-resistant ovarian cancer cell lines, we observed inhibition of cell proliferation only at the IC_50_ concentration, following long-term exposure [54]. In another cancer study, VIS induced a concentration-dependent effect on the proliferation of a gastric cancer cell line [69], colon cancer cell lines Caco-2 and Ht-29 [65], prostate cancer cell lines [70,71], and glioma cell lines [72].

The colony formation assay is a gold standard for investigating the long-term effects of compounds on cancer cell survival, colony formation potential, and the ability to divide indefinitely. At both concentrations used (IC_25_ and IC_50_), LDE225 and VIS very effectively inhibited colony formation. In contrast, LDE225 did not influence colony formation in chronic myeloid leukemia, although it was used in nanomolar concentrations in that study [73]. Inhibition of colony formation by VIS at concentrations of 20 and 40 µM was observed in head and neck squamous cell carcinoma (HNSCC) [74]. VIS also effectively inhibited colony formation in glioma cell lines at concentrations of 12.5, 25, and 50 µM [72]. Similarly, in another study, we observed concentration-dependent inhibition of colony formation in drug-sensitive and TOP-resistant ovarian cancer cell lines at concentrations of 10, 25, and 50 µM [54]. Thus, the ability of both inhibitors to inhibit colony formation in ovarian cancer indicates their potential as anticancer drugs.

Summarizing this 2D part of the study, we conclude that both inhibitors exerted both short-term and, most importantly, long-term impact on ovarian cancer cell proliferation. Furthermore, PAC-resistant cell lines were a few-fold more sensitive to both SMO inhibitors, although no synergistic effect of inhibitors with PAC on cell viability was observed. The 2D model of drug resistance is optimal for studying the mechanisms of drug resistance at the molecular and cellular levels. However, tumors are more complex structures, expressing ECM molecules and having a dense cellular structure that limits drug diffusion and induces CAM-DR, resulting in higher resistance to cytotoxic drugs [14,21]. Since we previously observed much higher resistance to cytotoxic drugs in the same cell lines grown as 3D spheroids, compared to 2D monolayers [6,7,23,51,75,76,77], we decided to investigate the response to SMO inhibitors in a 3D model that better reflects tumor conditions.

To better characterize our 3D model, we first compared the expression of drug-resistance proteins in the same cell lines grown under 2D and 3D culture conditions. Using Western blot, we observed very similar expression levels of P-gp, ALDH1A1, and COL3A1 under both conditions, consistent with our previous study, where we used the qPCR and IHC methods [23]. The expression of the Shh pathway genes also did not differ significantly between 2D and 3D cultures. In contrast, all cell lines grown as 3D spheroids showed decreased pTyr levels, consistent with literature data [78].

Subsequently, we investigated the response to both SMO inhibitors in 3D spheroids. In contrast to the 2D study, the differences in IC_50_ values between the drug-sensitive W1 and PAC-resistant cell lines seem to be diminished for both inhibitors, with the exception of VIS in the W1PR2. The differences in potency between LDE225 and VIS were higher in 3D spheroids than in the 2D model, with over 10-fold higher IC_50_ values for VIS than for LDE225 in the respective cell lines.

Direct comparison of IC_50_ values in the same cell lines grown as 2D monolayers and 3D spheroids indicated a stronger increase in resistance to LDE225 in drug-resistant cell lines compared to the W1 cell line. Similarly, a stronger increase in resistance to VIS was observed in the W1PR1 and the W1PR1-C7, compared to the W1 cell line.

Unfortunately, we could not directly compare our results with those of other researchers, as we found no studies comparing the response to LDE225 or VIS in the same cell lines grown under both 2D and 3D conditions. In our previous study using another ovarian cancer cell line, A2780, we observed only an approximately two-fold increase in resistance to VIS between 2D monolayers and 3D spheroids [54]. In the present study, the increase was three-fold in the W1 cell line. As both the W1 and the A2780 formed dense spheroids, these findings suggest that a dense cellular structure is not an effective barrier to VIS penetration. Although no other studies directly comparing LDE225 and VIS in 3D spheroids were identified, LDE225 at concentrations of 1–10 µM inhibited the formation and viability of spheroids formed by human prostate cancer stem cells [44]. Similarly, VIS inhibited spheroid formation by pancreatic cancer stem cells at 10 µM [59] and neurosphere formation at 50 µM [57]. Cyclopamine, another Shh inhibitor, also inhibited the growth of spheroids formed by different ovarian cancer cell lines [79].

However, we can compare the effects of LDE225 and VIS with those of cytotoxic drugs previously studied in this model [6,23]. Previously, we observed that the W1 and PAC-resistant cell lines form different kinds of spheroids. The drug-sensitive W1 cell line formed spherical, very dense spheroids and showed an enormous increase in resistance to cytotoxic drugs like PAC, MTX, or VIS between 2D and 3D conditions (about 3000-fold). In contrast, PAC-resistant cell lines formed elongated, low-density spheroids and showed only a 2–3-fold increase in resistance to PAC. These findings indicated that the dense spheroid structure in the W1 cell line effectively affects drug resistance. However, unlike cytotoxic drugs, LDE225 and VIS showed only a few-fold increase in resistance between 2D and 3D conditions in the W1, indicating that the dense spheroid structure itself is not an effective barrier to their penetration. Similar observations were previously reported in our study on VIS [54]. This suggests that both inhibitors can reach cancer cells within the dense tumor structures.

On the other hand, higher resistance to both SMO inhibitors in low-density 3D spheroids of drug-resistant cell lines indicates that mechanisms other than cell density may contribute to increased SMO inhibitor resistance in 3D. As we observed similar levels of Shh pathway gene expression in 2D and 3D models, these differences probably did not result from altered Shh pathway activity. Among other mechanisms influencing drug resistance in 3D models, interactions between cancer cells and ECM molecules through CAM-DR can increase cell survival by inducing pro-survival pathways and inhibiting apoptosis [13,15]. In W1PR1 and W1PR1-C7, we observed a similar increase in resistance to both LDE225 and VIS under 2D and 3D conditions, which may be explained by the increased role of CAM-DR. However, W1PR2 showed a strong increase in resistance to LDE225 (16-fold) but only a minor increase in resistance to VIS (2-fold). One possible explanation is that the inhibitors have different effects on intracellular COL3A1 expression. Although both inhibited COL3A1 expression in the cell culture medium of W1PR2, VIS was more effective in reducing intracellular COL3A1 expression. These differences may also result from different physicochemical properties of LDE225 and VIS, which require further investigation.

Next, we investigated the effect of both inhibitors on spheroid viability using CA-AM/PI staining. Calcein-AM (CA-AM) is a non-fluorescent, electrically neutral molecule that crosses the cell membrane and is cleaved by esterases in live cells, producing fluorescent calcein [80]. Thus, calcein labels live cells, whereas PI cannot diffuse through live cell membranes and mainly labels necrotic cells [81]. This double staining method has been used to measure spheroid viability after treatment with different compounds [82]. As we mentioned earlier, the W1 cell line formed high-density spheroids, whereas PAC-resistant cell lines formed low- to medium-density spheroids. These differences were reflected by the presence of a natural necrotic core exclusively in the W1 spheroids. Similar natural necrotic cores were observed by our team previously in A2780 ovarian cancer cell line spheroids [54]. Such necrotic cores result from high cell density limiting nutrient and gas diffusion [83,84]. In contrast, PAC-resistant cell line spheroids did not develop natural necrotic cores. Treatment of W1 spheroids with the inhibitors resulted in relaxation of spheroid structure, decreased CA staining, and increased PI staining. A more prominent effect was observed in low-density spheroids, where the increase in SMO inhibitor concentration resulted in decreased CA staining and a clear increase in PI staining. Thus, both SMO inhibitors induced concentration-dependent spheroid relaxation and cancer cell death.

In conclusion, we compared the effects of two SMO antagonists, LDE225 and VIS, in drug-sensitive and PAC-resistant ovarian cancer cell lines using 2D and 3D culture models. Direct comparison showed that LDE225 had a few-fold lower IC_50_ than VIS in all investigated cell lines. In 2D monolayers, drug-resistant cell lines were more sensitive to both inhibitors than the drug-sensitive W1 cell line, although these differences were less pronounced in 3D spheroids. Both inhibitors effectively inhibited proliferation and colony formation, processes related to cancer progression. The relatively small differences in IC_50_ values between 2D and 3D culture conditions in dense spheroids suggest that high cellular density does not prevent the activity of either inhibitor, which is a desirable feature of anticancer drugs. Our results suggest that LDE225 and VIS are promising candidates for ovarian cancer treatment. However, continued experiments using more complex models, such as CAM (chick embryo chorioallantoic membrane) or mouse models, are necessary to determine their therapeutic potential.

## 4. Materials and Methods

### 4.1. The Cell Culture Reagents and Antibodies

The reagents necessary for cell culture media preparation: RPMI 1640 media, fetal bovine serum (FBS), antibiotic-antimycotic solution (containing amphotericin, penicillin, and streptomycin), and L-glutamine were obtained from Corning (Corning, NY, USA). The antimycotic agent MycoZap™ Prophylactic was purchased from Lonza (Basel, Switzerland). Reagents used for cell splitting: trypsin-EDTA solution and DPBS (w/o Ca and Mg) were purchased from Corning (Corning, NY, USA). The cytotoxic drug, paclitaxel (PAC), used throughout the experiments, was purchased from Selleckchem (Huston, TX, USA). Primary antibodies used for immunofluorescence and Western blot procedures were as follows: ALDH1A1 monoclonal antibody (ab52492), obtained from Abcam (Cambridge, UK), Collagen Type III polyclonal antibody (22734-1-AP), bought from Proteintech (Rosemont, IL, USA), P-glycoprotein monoclonal antibody (ALX-801-002), purchased from Enzo (Farmingdale, NY, USA), Phosphotyrosine (p-Tyr) monoclonal antibody (A01819), obtained from GenScript (Nanjing, China), Beta Actin monoclonal antibody (66009-1-Ig) and GAPDH monoclonal antibody were bought from Proteintech (Rosemont, IL, USA). The secondary antibodies used in the immunofluorescence, Alexa Fluor^®^ 488 AffiniPure™, Anti-Rabbit (711-545-152) and Anti-Mouse (715-545-150), were acquired from Jackson ImmunoResearch (West Grove, PA, USA). The secondary antibodies used in Western blot, Anti-rabbit HRP antibody (SA00001-2) and Anti-mouse HRP antibody (SA00001-1-A), were bought from Proteintech (Rosemont, IL, USA). SMO inhibitors, LDE225 and Vismodegib, were purchased from Molnova (Ann Arbor, MI, USA).

### 4.2. Cell Culture

The human ovarian cancer cell line W1 was used in all of the experiments. It was first established from tissue obtained from an untreated ovarian cancer patient, and the sublines resistant to paclitaxel (W1PR1 and W1PR2) were later generated by exposing the W1 cell line to increasing concentrations of PAC [53]. Subsequently, the ALDH1A1-negative cell line W1PR1C7 was obtained by gene knockout in the W1PR1 cell line [51]. All the cell lines were cultured in RPMI 1640 medium supplemented with 10% (*v*/*v*) fetal bovine serum, 2 mM L-glutamine, and the following antibiotic concentrations: 100 U/mL streptomycin, 0.1 mg/mL penicillin, and 25 µg/mL amphotericin B. Additionally, the antimycotic compound MycoZap™ was added to the media in the final dilution of 1:1000. The incubation conditions were a 5% CO_2_ atmosphere and 37 °C.

### 4.3. RT-qPCR Workflow Reagents

RNA was extracted using the Gene Matrix Universal RNA Purification Kit (EURx, Ltd., Gdańsk, Poland), and the concentration of the obtained material was measured by checking the absorbance at 260 nm and 280 nm. Afterwards, the cDNA was synthesized using 1.5 μg of RNA, 1 µL of oligodT18A primer (IBB PAN, Warsaw, Poland), and 1 µL of dNTP mix (Thermo Fisher, Waltham, MA, USA, R0192) for each sample. The samples underwent denaturation at 65 °C for 5 min, then the following reagents necessary for reverse transcription were added: 4 µL of 5× First Strand buffer, 2 µL of DTT, 0.5 µL of RNaseOUT RNase inhibitor, and 0.5 µL of M-MLV RT reverse transcriptase. The reaction conditions were 60 min at 37 °C, followed by 15 min at 75 °C.

Afterwards, the real-time PCR reaction mixture was prepared, with every test consisting of 12.5 μL of Takyon™ ROX SYBR^®^ MasterMix blue dTTP (Eurogentec, Searing, Belgium), 1 μL of the forward primer, 1 μL of the reverse primer (each pair of primers was sequence-specific and the concentration of primers used was 7.5 μM) provided by Oligo.pl (Warsaw, Poland) (Table 8), 9.5 μL of nuclease-free water, and 1 μL of cDNA solution. Nuclease-free water was used instead of the cDNA in the negative control. GAPDH was the reference gene. Amplification was carried out with a CFX Opus 96 Real-Time PCR system (Bio-Rad Laboratories, Hemel Hempstead, UK), programmed as follows: initial denaturation at 95 °C for 15 min, then 45 cycles composed of subsequent denaturation, annealing and extension (denaturation at 95 °C for 15 s, annealing at 60 °C for 30 s, and extension at 72 °C for 30 s), followed by a final extension at 72 °C for 30 s.

### 4.4. Two-Dimensional MTT Assay

The cells were seeded into 96-well plates at a density of 3 × 10^3^ cells per well, suspended in 200 μL of cell culture media. After 48 h, the medium was replaced with the cytotoxic drugs and/or inhibitors, diluted in media in proper concentrations. Following a 72-h incubation, the medium was removed, and the MTT test was performed. The Thiazolyl Blue powder was diluted in PBS to a concentration of 5 mg/mL, and 20 μL of it was added to each well, along with 150 μL of cell culture media, making the final concentration of 0.59 μg/mL. Then, following a 1-h incubation, the media were replaced with 200 μL of DMSO to dissolve the formazan crystals. Then, the absorbance in each well was measured at 570 nm and 720 nm with a Synergy LX Multi-Mode Reader (BioTek Instruments, Inc., Winooski, VT, USA). Every experiment was done in duplicate and repeated at least 4 times. Based on the results obtained, response curves were generated from the absorbance measurements. The absorbance of untreated control cells was set at 100%, and the absorbance measured in cells treated with the respective drugs and/or inhibitors was expressed as a percentage of the control. IC_50_ values were determined by linear regression based on the response curves.

### 4.5. Three-Dimensional MTT Assay

The cell lines were seeded into non-adherent 96-well plates (BRAND plates inertGrade, F-bottom, 781902, Merck, Darmstadt, Germany) at a density of 1 × 10^4^ cells per well, suspended in 200 μL of cell culture media. After 48 h, 100 μL of the medium was replaced with the cytotoxic drugs and/or inhibitors, diluted in media to achieve the proper final concentrations. Following a 72-h incubation, 100 μL of medium was removed, and the MTT test was performed with the Cell Proliferation Kit I (Roche, cat. no. 11465007001)—10 μL of MTT reagent was added to achieve the final concentration of 0.45 µg/mL. Following a 4-h incubation, 100 μL of the solubilization buffer was added for an overnight incubation at 37 °C. Then, the absorbance in each well was measured at 570 nm and 720 nm with a Synergy LX Multi-Mode Reader (BioTek Instruments, Inc.). Every experiment was done in duplicate and repeated at least 4 times. Based on the results obtained, response curves were generated from the absorbance measurements. The absorbance of untreated control cells was set at 100%, and the absorbance measured in cells treated with the respective drugs and/or inhibitors was expressed as a percentage of the control. IC_50_ values were determined by linear regression based on the response curves.

### 4.6. Immunofluorescence

The coverslips were placed into 24-well plates, and then a suspension of 2.5 × 10^4^ cells in 500 μL of medium was added for each tested cell line. After reaching approx. 70% confluence, the cells were fixed and permeabilized with chilled 1:1 acetone/methanol for 15 min at RT. Afterwards, the cells were blocked at RT with 3% BSA for 30 min. The next step was a 2-h incubation with the primary antibody, followed by a 1-h incubation with the secondary antibody. Lastly, the coverslips were washed with demineralized water and glued to the slides with Fluoroshield™ with DAPI mounting solution (Merck, Darmstadt, Germany). The coverslips were washed with PBS three times before each step. The prepared slides were then observed, and the cells were photographed under a fluorescent microscope (Zeiss AxioImager.Z1, Oberkochen, Germany) with Zen Blue v3.3 software.

For the analysis of COL3A1 fluorescence following LDE225 and VIS treatment, a suspension of 1 × 10^4^ cells in 500 μL of medium was added to the 24-well plates with inserted coverslips for each tested cell line. After 48 h, the medium was replaced with medium with diluted inhibitors at appropriate concentrations and incubated for another 72 h. Next, the cells were fixed, and the slides were prepared, following the protocol described above.

### 4.7. Protein Isolation and Western Blot Analysis

Cell lines were plated in 6-well plates at a density of 2 × 10^5^ cells per well in 2000 µL of culture medium. After 48 h, the medium was replaced with inhibitor-free medium or with medium containing the proper inhibitor at selected concentrations. Following 72 h of incubation, total cellular protein or protein from the medium was extracted.

For total cellular protein extraction, the process began with the cells being washed three times with PBS supplemented with Ca^2+^ and Mg^2+^ ions and lysed in RIPA buffer containing a protease inhibitor cocktail and PhosSTOP phosphatase inhibitor (Roche Diagnostics GmbH, Mannheim, Germany) for 80 min at 4 °C. Lysates were subsequently centrifuged at 13.4 × 10^3^ rpm for 30 min at 4 °C, and protein concentration was quantified using the Bradford assay (Bio-Rad Laboratories, Hemel Hempstead, UK).

To isolate protein from the medium, 10 mL of medium was collected for every analyzed condition, and then the medium was concentrated using Amicon Centrifugal concentrators with Ultracel^®^ membrane,3 kDa MWCO (Amicon, Millipore UFC9003, Burlington, MA, USA), according to the standard manufacturer’s protocol. Next, the protease inhibitor cocktail and PhosSTOP phosphatase inhibitor (Roche Diagnostics GmbH, Mannheim, Germany) were added to the concentrates, and protein concentration was quantified using the Bradford assay (Bio-Rad Laboratories, Hemel Hempstead, UK).

Protein samples (7 µg) were mixed with 4× loading buffer and 10% β-mercaptoethanol, then resolved by SDS-PAGE on 4–20% Mini-PROTEAN^®^ TGX™ gels. Separated proteins were transferred onto nitrocellulose membranes, except for pTYR detection in the inhibitor-free experiment, where PVDF membranes were used. Transfer was performed using the Trans-Blot^®^ system (Bio-Rad). Membranes were blocked for 1 h in either 5% BSA (for pTYR) or 5% non-fat milk in TBS-T (0.1 M Tris-HCl, 0.15 M NaCl, 0.1% Tween 20). Primary antibody incubation was carried out overnight using anti-ALDH1A1 (1:2000), anti-P-gp (1:400), anti-COL3A1 (1:1500)—all of which were diluted in 5% non-fat milk in TBS-T, or anti-pTYR (1:1000, diluted in 5% BSA). After washing, membranes were incubated for 3 h with a proper HRP-conjugated goat secondary antibody (1:7500). Protein bands were visualized using an enhanced chemiluminescence detection system (ECL, Femto Super Signal Reagent) and imaged with ChemiDoc™ (Bio-Rad Laboratories). Subsequently, membranes were washed and reprobed with antibodies against β-actin (1:10,000) or GAPDH (1:30,000), followed by incubation with HRP-conjugated secondary antibody under the same conditions. Signals were again detected using ECL and ChemiDoc™.

### 4.8. Assessment of Colony Formation

The cell line suspensions were seeded into 6-well plates at a density of 500 cells per well. Following 48-h incubation, the medium was replaced with medium containing selected concentrations of the inhibitors. Next, after 72 h of incubation, the medium was replaced once again with fresh medium containing the same inhibitor concentrations. Finally, after 4 days of additional incubation, the cells were rinsed with PBS supplemented with Mg^2+^ and Ca^2+^, and then fixed in methanol for 15 min. Then, the colonies were stained with crystal violet (0.5% in 20% methanol) for 15 min. The dye was then washed away with running tap water and left to dry. The plates were scanned, and ImageJ software (1.48q, Rayne Rasband, National Institutes of Health, USA)was used to calculate the area occupied by the colonies.

### 4.9. Cell Proliferation Assay

Cell lines were plated into several 96-well plates at a density of 1 × 10^3^ cells per well in 200 µL of culture medium. After 48 h, the medium in all of the plates was replaced with inhibitor-free medium or with medium containing the proper inhibitor at selected concentrations. The MTT assay was performed on a plate labeled as the 0-h point. Subsequent MTT assays were conducted on the other plates, one every 24 h for 4 days, allowing determination of cell line proliferation relative to the 0-h point. The experiment was repeated at least four times.

### 4.10. Spheroid Viability Assay

The cell lines were seeded into non-adherent 96-well plates (BRAND plates inertGrade, F-bottom, 781902, Merck, Darmstadt, Germany) at a density of 7.5 × 10^4^ cells per well, suspended in 200 μL of cell culture media. After 48 h, 100 μL of the medium was replaced with the inhibitors, diluted in the medium to achieve the desired final concentrations. Following a 72-h incubation, 100 μL of the medium was again removed and replaced with the solution of fluorescent agents, achieving final concentrations of 1 µM elacridar, 0.5 μM Calcein-AM, and 10 μg/mL Propidium Iodide in each test sample. Following a 2-h incubation, the spheroids were washed 4 times with a 50 μM Verapamil solution in PBS by removing 100 μL of medium and adding the same volume of the solution to block ABC transporters during observation. Immediately afterwards, the aggregates were observed and photographed under the Leica DMI8 microscope (Leica, Wetzlar, Germany), with Leica Application Suite X (Leica Microsystems GmbH, Wetzlar, Germany) Software, LAS X 5.3.3.

### 4.11. Statistical Analysis

The results obtained from the qPCR and MTT assays were statistically evaluated using Student’s *t*-test and expressed as the mean ± standard error of the mean (SEM). Statistical significance was established at a *p*-value < 0.05. All experiments were performed using at least three independent replicates. IC_50_ values were calculated based on data from at least four independent experiments. In each experiment, two technical replicates were plated, and the mean value of the technical replicates was used for IC_50_ estimation. The minimum and maximum IC_50_ values obtained for each cell line and experimental condition are reported in parentheses in the respective tables.

## Figures and Tables

**Table 1:** Summary of LDE225 sensitivity in W1 and paclitaxel-resistant sublines cultured under 2D conditions. Mean IC_25_ and IC_50_ values are presented for each cell line, with the corresponding ranges shown in parentheses. The W1 cell line was used as the reference (relative resistance = 1). Underlined values indicate fold changes in sensitivity relative to W1 cells, while arrows indicate decreased (↓) IC_50_ values compared with the parental cell line. Statistical significance was assessed using Student’s *t*-test, *** *p* < 0.001.

Cell Line	IC_25_ [µM]	IC_50_ [µM]
W1	3.39 (3.03–3.43) 1	5.03 (4.78–5.15) 1
W1PR1	0.52 (0.38–0.75) 6.62 ↓***	1.27 (0.85–1.82) 3.96 ↓***
W1PR1-C7	0.66 (0.54–0.87) 5.13 ↓***	1.45 (1.00–1.85) 3.47 ↓***
W1PR2	0.31 (0.18–0.46) 10.3 ↓***	0.76 (0.67–0.90) 6.62 ↓***

**Table 2:** Summary of Vismodegib sensitivity in W1 and paclitaxel-resistant sublines cultured under 2D conditions. Mean IC_25_ and IC_50_ values are presented for each cell line, with the corresponding ranges shown in parentheses. The W1 cell line was used as the reference (relative resistance = 1). Underlined values indicate fold changes in sensitivity relative to W1 cells, while arrows indicate decreased (↓) IC_50_ values compared with the parental cell line. Statistical significance was assessed using Student’s *t*-test, ** *p* < 0.01 and *** *p* < 0.001.

Cell Line	IC_25_ [µM]	IC_50_ [µM]
W1	17.33 (15.67–19.05) 1	33.90 (32.33–35.56) 1
W1PR1	6.50 (6.30–6.99) 2.67 ↓**	11.1 (10.51–12.76) 3.05 ↓***
W1PR1-C7	6.44 (5.18–7.61) 2.69 ↓**	11.11 (9.32–13.7) 3.05 ↓***
W1PR2	5.19 (4.61–5.58) 3.34 ↓**	10.3 (8.99–11.42) 3.29 ↓***

**Table 3:** Summary of paclitaxel (PAC) sensitivity in W1 and paclitaxel-resistant sublines cultured under 2D conditions with or without LDE225 at IC_10_, IC_25_, and IC_50_ concentrations (0.25, 0.5, and 1 μM for W1PR1 and W1PR1-C7 cells; 0.1, 0.3, and 0.6 μM for W1PR2 cells). Mean PAC IC_50_ values are presented for each cell line. Cells treated with PAC alone were used as the reference (relative resistance = 1). Underlined values indicate fold changes in sensitivity relative to cells treated with PAC alone, while arrows indicate increased (↑) or decreased (↓) IC_50_ values compared with the control.

Cell Line	Control PAC IC_50_ (ng/mL)	LDE225 IC_10_ PAC IC_50_ (ng/mL)	LDE225 IC_25_ PAC IC_50_ (ng/mL)	LDE225 IC_50_ PAC IC_50_ (ng/mL)
W1PR1	1672 (1422–1929) 1	1865 (1314–2869) 1.15 ↑	1800 (1207–2679) 1.08 ↑	1233 (987–1375) 1.35 ↓
W1PR1-C7	1971 (1375–2627) 1	1929 (1415–2379) 1.02 ↓	1734 (1270–1967) 1.11 ↓	1543 (991–1821) 1.27 ↓
W1PR2	3796 (2320–5311) 1	3761 (1966–5465) 1.01 ↓	3660 (1925–4853) 1.03 ↓	3491 (2416–4659) 1.09 ↓

**Table 4:** Summary of paclitaxel (PAC) sensitivity in W1 and paclitaxel-resistant sublines cultured under 2D conditions with or without Vismodegib (VIS) at IC_10_, IC_25_, and IC_50_ concentrations (2, 5, and 10 μM). Mean PAC IC_50_ values are presented for each cell line. Cells treated with PAC alone were used as the reference (relative resistance = 1). Underlined values indicate fold changes in sensitivity relative to cells treated with PAC alone, while arrows indicate increased (↑) or decreased (↓) IC_50_ values compared with the control.

Cell Line	Control PAC IC_50_ (ng/mL)	VIS IC10 PAC IC_50_ (ng/mL)	VIS IC25 PAC IC_50_ (ng/mL)	VIS IC_50_ PAC IC_50_ (ng/mL)
W1PR1	1866 (1439–2285) 1	1872 (1473–2194) 1.00 ↑	1924 (1348–2260) 1.03 ↑	1622 (1192–1880) 1.15 ↓
W1PR1-C7	1965 (1374–2664) 1	1986 (1351–2679) 1.01 ↓	1970 (1410–2230) 1.00 ↑	1225 (776–1705) 1.6 ↓
W1PR2	3098 (1998–3961) 1	3508 (2425–4469) 1.13 ↑	3027 (1870–4037) 1.02 ↓	1768 (1131–2411) 1.75 ↓

**Table 5:** Summary of LDE225 sensitivity in W1 and paclitaxel-resistant sublines cultured under 3D conditions. Mean IC_50_ values are presented for each cell line, with the corresponding ranges shown in parentheses. The W1 cell line was used as the reference (relative resistance = 1). Underlined values indicate fold changes in sensitivity relative to W1 cells, while arrows indicate decreased (↓) IC_50_ values compared with the parental cell line. * *p* < 0.05.

Cell Line	IC_50_ [µM]
W1	14.32 (11.1–16.3) 1
W1PR1	8.06 (5.00–11.4) 1.78 ↓*
W1PR1-C7	12.9 (9.88–16.44) 1.11 ↓
W1PR2	12.15 (10.39–13.62) 1.18 ↓

**Table 6:** Summary of Vismodegib (VIS) sensitivity in W1 and paclitaxel-resistant sublines cultured under 3D conditions. Spheroids were cultured with or without VIS for 72 h. Mean IC_50_ values are presented for each cell line, with the corresponding ranges shown in parentheses. The W1 cell line was used as the reference (relative resistance = 1), and for the remaining cell lines, values are expressed as multiples of the sensitive control. Underlined values indicate fold changes in sensitivity relative to cells treated with VIS alone, while arrows indicate decreased (↓) IC_50_ values compared with the control. ** *p* < 0.01.

Cell Line	IC_50_ [µM]
W1	183 (90.2–300) 1
W1PR1	110 (45.6–175) 1.67 ↓
W1PR1-C7	130 (46.6–183) 1.41 ↓
W1PR2	25.1 (10.7–43.1) 7.33 ↓**

**Table 7:** Summary of cell line resistance to SMO inhibitors (LDE225 and Vismodegib) in 2D and 3D models. Mean IC_50_ values for SMO inhibitors were determined for each cell line, with the corresponding ranges shown in parentheses. Monolayer (2D) cultures of each cell line were used as the reference (relative resistance = 1, underlined), while three-dimensional (3D) cultures were expressed as fold changes relative to the corresponding 2D conditions, underlined. Arrows indicate increased (↑) IC_50_ values compared with monolayer cultures. * *p* < 0.05, ** *p* < 0.01, *** *p* < 0.001.

Cell Line	IC_50_ [µM] 2D	IC_50_ [µM] 3D
**LDE225**		
W1	5.03 (4.78–5.15) 1	14.32 (11.1–16.3) 2.85 ↑**
W1PR1	1.27 (0.85–1.82) 1	8.06 (5.00–11.4) 6.34 ↑*
W1PR1-C7	1.45 (1.00–1.85) 1	12.9 (9.88–16.44) 8.85 ↑**
W1PR2	0.76 (0.67–0.90) 1	12.15 (10.39–13.62) 16.0 ↑***
**Vismodegib**		
W1	33.90 (32.33–35.56) 1	183 (90.2–300) 5.42 ↑**
W1PR1	11.1 (10.51–12.76) 1	110 (45.6–175) 9.89 ↑**
W1PR1-C7	11.11 (9.32–13.7) 1	130 (46.6–183) 11.7 ↑*
W1PR2	10.3 (8.99–11.42) 1	25.1 (10.7–43.1) 2.43 ↑**

**Table 8:** Primer sequences used for qPCR analysis.

Transcript	Sequence (5′−3′ Direction) Forward	Sequence (5′−3′ Direction) Reverse	ENST Number http://www.ensembl.org	Product Size (bp)
ALDH1A1	GTTGTCAAAC CAGCAGAGCA	CTGTAGGCCC ATAACCAGGA	00000165092	115
COL3A1	AAGGTCCAGC TGGGATACCT	CACCCTTTAA TCCAGGAGCA	00000304636	105
GAPDH	GAAGGTGAAG GTCGGAGTCA	GACAAGCTTC CCGTTCTCAG	00000229239	199
GLI1	ACACGGGTGA GAAGCCATAC	GCAGCCAGGG AGCTTACATA	00000228682	134
GLI2	TGGAGCACTA CCTCCGTTCT	CCCCTCTCCT TAAGGTGCTC	00000361492	109
MDR1	TGACAGCTAC AGCACGGAAG	TCTTCACCTC CAGGCTCAGT	00000265724	131
PCTCH1	GGCACAGTCA AGAACAGCAC	TGTCCTCGTT CCAGTTGATG	00000430669.6	139
SHH	TCCAAGGCAC ATATCCACTG	CTCAGGTCCT TCACCAGCTT	00000297261.7	128
SMO	TACGTCAATGC GTGCTTCTT	CGCAGGACAG AGTCTCATTG	00000249373.8	134

## Data Availability

The data presented in this study are openly available in [https://repod.icm.edu.pl/dataset.xhtml?persistentId=doi:10.18150/BRZG6Y], accessed on 29 June 2026, and at [doi.org/10.18150/BRZG6Y].
